# Aggregation-induced phosphorescence sensitization in two heptanuclear and decanuclear gold–silver sandwich clusters†

**DOI:** 10.1039/d0sc05095d

**Published:** 2020-10-30

**Authors:** Zhou Lu, Yu-Jie Yang, Wen-Xiu Ni, Mian Li, Yifang Zhao, Yong-Liang Huang, Dong Luo, Xiaoping Wang, Mohammad A. Omary, Dan Li

**Affiliations:** College of Chemistry and Materials Science, Guangdong Provincial Key Laboratory of Functional Supramolecular Coordination Materials and Applications, Jinan University Guangzhou 510632 P. R. China danli@jnu.edu.cn; Department of Chemistry, University of North Texas 1155 Union Circle #305070 Denton Texas 76203 USA omary@unt.edu; Department of Chemistry, Shantou University Guangdong 515063 P. R. China; Department of Chemistry, Shantou University Medical College Shantou Guangdong 515041 P. R. China; Neutron Scattering Division, Oak Ridge National Laboratory Oak Ridge Tennessee 37831-6475 USA

## Abstract

The strategy of aggregation-induced emission enhancement (AIEE) has been proven to be efficient in wide areas and has recently been adopted in the field of metal nanoclusters. However, the relationship between atomically precise clusters and AIEE is still unclear. Herein, we have successfully obtained two few-atom heterometallic gold–silver hepta-/decanuclear clusters, denoted Au_6_Ag and Au_9_Ag, and determined their structures by X-ray diffraction and mass spectrometry. The nature of the Au^I^⋯Ag^I^ interactions thereof is demonstrated through energy decomposition analysis to be far-beyond typical closed-shell metal–metal interaction dominated by dispersion interaction. Furthermore, a positive correlation has been established between the particle size of the nanoaggregates and the photoluminescence quantum yield for Au_6_Ag, manifesting AIEE control upon varying the stoichiometric ratio of Au : Ag in atomically-precise clusters.

## Abstract 2

The strategy of aggregation-induced emission enhancement (AIEE) has been proven to be efficient in wide areas and has recently been adopted in the field of metal nanoclusters.

## Graphical abstract

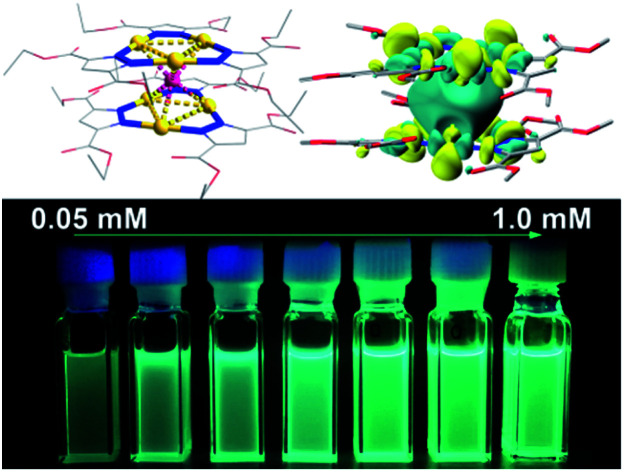

## Introduction

Synthesis of few-atom nanoclusters (NCs) is a work of art and represents the atomic precision of chemistry, building a bridge from traditional coordination chemistry to plasmonic metal clusters.^1–5^ With advanced single crystal X-ray analysis and high-quality mass spectrometry techniques at hand, precise formulas and total structures of both cluster kernels and protecting ligands can be obtained to facilitate the exploration of the structure–property relationship at the molecular scale.^6,7^ Nevertheless, achieving a unanimous interpretation of metallophilicity (aka. metal–metal interaction) in fields spanning from oligomeric metal complexes to atomically precise metal NCs remains a Sisyphus task by means of multiple descriptions in different narratives.^1,2,8–18^

So far, the majority of research studies in these fields have focused on same-atom clusters rather than heterometallic ones due to the synthetic challenge of controllable doping/tailoring of another kind of atom at the level of atomic precision.^5,7,14,19^ One of the topical studies is the doping of silver atoms into gold clusters, whereby their same valence shell electrons and similar coordination modes could bring unique optoelectronic behaviours.^20–26^ A representative example is gradually substituting gold atoms with silver in the 25-metal-atom NC Ag_*x*_Au_25−*x*_ and the 13th silver atom replacement results in a 200-fold boost in photoluminescence quantum yield (*Φ*_PL_)^20^ which is attributed to a combination of stabilization of the lowest unoccupied molecular orbital (LUMO), rigidity enhancement, and symmetry preservation despite the great perturbation of the electronic structure.^21^ In other words, the stoichiometric ratio of Au : Ag is a key factor in regulating the photophysics of these molecular clusters.^22–26^

The aggregation of molecules in solution is a ubiquitous phenomenon in supramolecular and biological systems and has been recognized as a promising strategy to enhance the photoluminescence of ordered nanostructures by virtue of metal–metal interactions/bondings,^27–30^ sometimes accompanied by other non-covalent interactions among the protecting ligands (*e.g.* π–π/C–H⋯π interactions, hydrogen bonding).^31^ The concept of aggregation-induced emission (AIE), first discovered in a class of organic molecules undergoing a mechanism of restriction of intramolecular motions (RIM) to boost luminescence,^32^ is now relegated to a phenomenal description propagating broadly to many areas, such as metal complexes,^33,34^ supramolecular cages^35,36^ and self-assembled metal NCs.^27,37–44^ It is unsurprising that a mist steals over the comprehension of AIE in NCs given the deadlock of even more blurred structure–property correlation compared with the aforementioned.

Previously, we have demonstrated the self-assembly of a cyclic trinuclear Au^I^–pyrazolate complex and silver cation in solution or by mechanochemistry approaches and managed to determine the single crystallographic structure of a heptanuclear sandwich-like cluster.^45^ Herein, we report the synthesis, crystal structure, metal–metal bonding analyses, and unusual observation of stoichiometric-ratio-dependent aggregation-induced emission enhancement (AIEE) in the self-assembly processes of two atomically precise gold–silver clusters in solution.

## Results and discussion

**Scheme 1:**
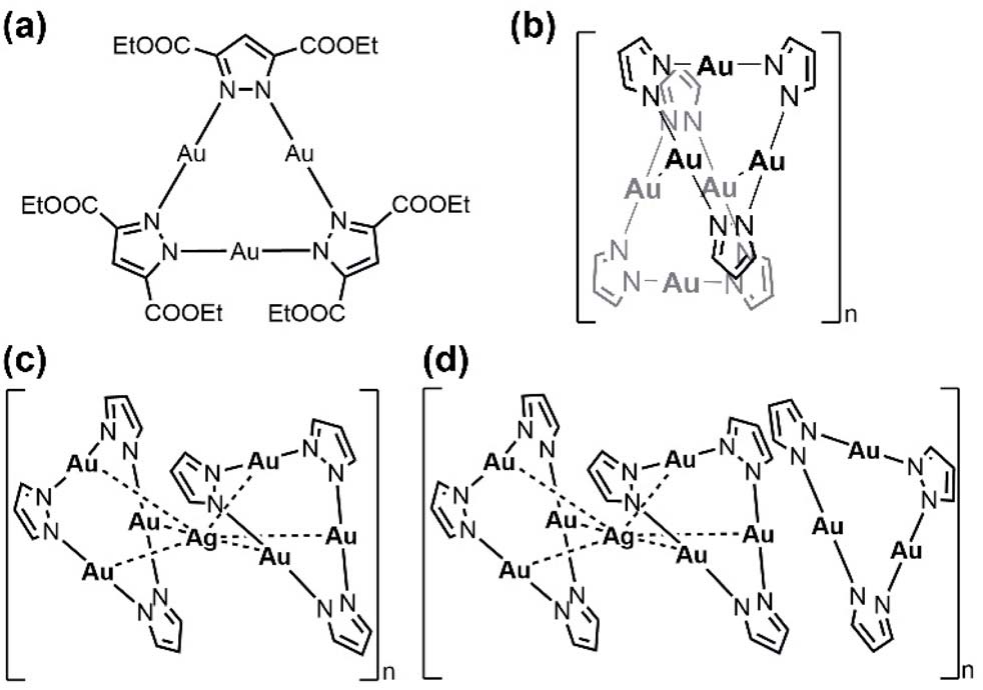
Chemical structures of (a) Au_3_ and (b) a dimer of Au_3_, and sandwich complexes (c) Au_6_Ag and (d) Au_9_Ag discussed in this work (ligand substituents and counteranions PF_6_^−^ are omitted from the figures for clarity).

**Fig. 1:**
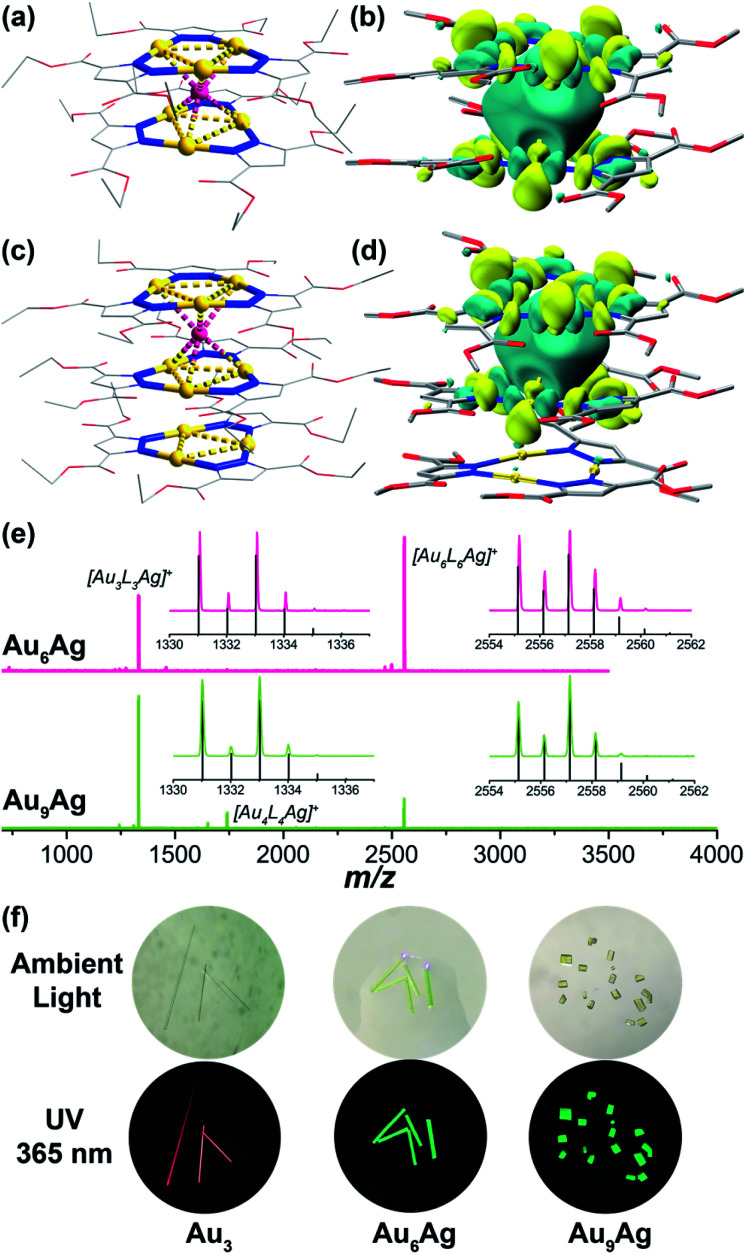
Crystal structures for (a) Au_6_Ag and (c) Au_9_Ag and molecular orbital contours of the LUMOs for (b) Au_6_Ag* and (d) Au_9_Ag* (*vide infra*, isovalue = 0.02). Hydrogens, counteranions PF_6_^−^, and disordered sets in the Au_3_ rings and Ag cations are omitted from the figures for clarity. (e) MALDI-TOF-MS spectra of (top) Au_6_Ag and (bottom) Au_9_Ag. Inset: simulated and measured isotopic distributions of (left) [Au_3_L_3_Ag]^+^ and (right) [Au_6_L_6_Ag]^+^ fragments. (f) Digital photographs of crystals of Au_3_, Au_6_Ag and Au_9_Ag under ambient and 365 nm UV light, respectively.

The cyclic trinuclear Au^I^ complex (denoted Au_3_, Scheme 1a) based on the ligand bis-3,5-(ethoxycarbonyl)_2_-1*H*-pyrazole (*H*L) was chosen as the precursor due to its unique near-planar geometry and aromatic/electron-rich property.^1,2,46^ In previous work done by Chilukuri, Omary, Hipps, and co-workers, Au_3_ showed one-dimensional chair-stacking led by two pairs of alternate intermolecular Au^I^⋯Au^I^ interactions (Scheme 1b, 3.273 Å & 3.493 Å).^47^ The reported routes to heterometallic NCs include co-reduction of two metal ion precursors, intercluster reactions and metal tailoring to homometallic NCs, which lack the prediction of the doping/alloying positions. Controllable syntheses at a precise atomic level have been occasionally reported by adopting a metal cluster as the precursor owing to abundant metal/ligand interacting sites, especially through the insertion of a silver cation into Au NCs.^14,48^ In the present work, solution-based reactions between Au_3_ and AgPF_6_ in different stoichiometric ratios afford two sandwich-like clusters [Au_3_–Ag^+^–Au_3_][PF_6_^−^] and {Au_3_–Ag^+^–[Au_3_]_2_}[PF_6_^−^] (denoted Au_6_Ag and Au_9_Ag, Scheme 1c and d) supported by Lewis-acid/π-base and cation–π interactions, restricting foreign ions along the *C*_3_ axis (Fig. 1).

X-ray photoelectron spectroscopy (XPS) studies proved the monovalence of the gold and silver atoms without reduction before or after the reactions (Fig. S5†). Single crystals suitable for X-ray structural analysis were obtained from gas-phase diffusion or slow evaporation under room temperature for needle-like Au_6_Ag and block-shaped Au_9_Ag, respectively. X-ray crystallographic studies reveal that both heterometallic Au^I^–Ag^I^ clusters exhibit infinite column stacking modes in one dimension (*c*-axis), containing sandwich-like [Au_3_–Ag^+^–Au_3_] fragments (Fig. 1a, c and S13–S15†). Au_6_Ag crystallizes in a *P*6̄ space group and Au_9_Ag crystallizes in a *P*6̄2*c* space group; Au_3_ species remain intact in both heterobimetallic clusters and follow the *C*_3_ rotation symmetry. Different from other ligand-unsupported Au–Ag clusters, the sole silver cation is totally surrounded by six Au atoms (not coordinated with organic ligands) to form a twisted trigonal prism; the distances of the Au–Ag contacts fall in the range of 2.617(5)–2.7871(7) Å (Table S2†), shorter than most ligand-supported/unsupported gold–silver contacts.^49^ Besides, in Au_9_Ag, we also find an additional Au_3_ in the repeating unit showing close intertrimer Au⋯Au distances of 3.175(6)–3.649(3) Å (Fig. 1c). The full-sandwich-like fragments ([Au_6_L_6_Ag]^+^, *m*/*z* = 2557.198 in Au_6_Ag; 2557.138 in Au_9_Ag) and half-sandwich fragments ([Au_3_L_3_Ag]^+^, *m*/*z* = 1333.060 in Au_6_Ag; 1333.009 in Au_9_Ag), as well as their resolved isotopic peaks, are confirmed by matrix-assisted laser desorption/ionization time-of-flight mass spectroscopy (MALDI-TOF-MS, Fig. 1e). The full triple-decker cluster of Au_9_Ag ([Au_9_L_9_Ag]^+^, calculated *m*/*z* = 3781.253) could not be observed in the MS study, probably due to the lability of the intermolecular Au^I^⋯Au^I^ interactions.

To evaluate the binding energies of ligand-unsupported Au^I^⋯Ag^I^ and Au^I^⋯Au^I^ interactions, density functional theory (DFT) calculations were carried out (ESI,† Computational section). In previous reports, the estimated energy of 14 kcal mol^−1^ was regarded as ligand-unsupported Au^I^⋯Ag^I^ metallophilicity and ground state charge-transfer character, behaving like “loose clusters”.^9^ Here, our energy decomposition analysis (EDA) reveals that electrostatic attraction and orbital interaction also make considerable contributions, suggesting that the interaction between the silver cations and Au_3_ is beyond Lewis acid/π-base or cation–π interactions (Fig. S28†). The orbital interaction value of ∼18 kcal mol^−1^ for each ligand-unsupported Au^I^⋯Ag^I^ interaction (six pairs of ∼110 kcal mol^−1^ in total, Table S9†) is regarded as strong (dative) bonding by the natural orbitals for chemical valence (NOCV) method with electron-sharing from gold to silver, along with considerable dispersion energy (Fig. S29–S33†).^8,18,50,51^ A recent report revealed that for metal–metal bonds of metals with filled d-orbitals, the charge-shift character increases as the covalency decreases.^52^ The electrostatic interactions lead to contributions of about 50% to the attraction energies in the EDA results, corresponding to the remarkable charge-shift character of the later transition metals. As a result, the synergic non-bonding interactions, including Lewis acid/π-base interactions (electrostatic derivation) and closed-shell metal–metal interactions (dispersion force), lead to Au–Ag bonding (orbital interactions) with remarkable bonding energies. Besides, the intertrimer Au^I^⋯Au^I^ interactions in Au_9_Ag are only reckoned as aurophilicity-mediated by relativistic effects, reflected by the other derivative dispersion energy.^53^ According to the molecular orbital analysis, the LUMOs of Au_6_Ag* and Au_9_Ag* (replacing ethoxycarbonyl with methoxycarbonyl groups due to little contribution to the electronic structures) are mainly composed of the Au 5d and Ag 5s atomic orbitals in the sandwich-like fragments (Fig. 1b and d), showing strong sd hybridizations attributed to relativistic contractions.^7,20,54^ The inserted silver cation only alters the electronic density of the sandwich-like fragments in Au_9_Ag*, with the additional Au_3_ hardly contributing to the LUMO.^45^ By contrast, the composition of the 5d_*z*^2^_ orbitals from all Au atoms could be identified in doubly-degenerate filled frontier orbitals (two highest-occupied molecular orbitals (HOMOs) and two next-HOMOs (HOMO−1s) – see Fig. S36–S37†). This indicates remarkable metal contribution to the frontier occupied molecular orbitals. The energy level diagram (Scheme S1†) clearly reflects the great perturbation of the inserted silver ions, rendering higher-density electron delocalization in the metal kernels, as well as more stabilized frontier virtual orbitals (LUMOs) and narrower HOMO–LUMO gaps.

Au_3_, Au_6_Ag, and Au_9_Ag are all strongly emissive in the solid state (Fig. 1f and S16†). The homometallic Au_3_ emits at the maximum of 670 nm with *Φ*_PL_ of 67.5% under room temperature; both heterobimetallic nanoclusters Au_6_Ag and Au_9_Ag show the maximum emission peak at 496 nm with *Φ*_PL_ of 43.9% and 12.0%, respectively. The room-temperature emission lifetimes fall into the microsecond region (*τ* = 15.32, 9.31, and 9.11 μs for Au_3_, Au_6_Ag, and Au_9_Ag, respectively; Table S5, Fig. S25 and S26†), characteristic of phosphorescence. Time-dependent density functional theory (TD-DFT) calculations reveal that the phosphorescence of Au_3_ originates from intra-ligand charge transfer (^3^ILCT) and metal-to-ligand charge transfer (^3^MLCT), despite the relatively short intermolecular Au⋯Au distances. In contrast, the added Au^I^⋯Ag^I^ bonding manifests as low-energy absorption (around 400 nm, Fig. S17†), consistent with the TD-DFT results of ligand-protected metal centred (^1^MC/^3^MC) characteristics (Fig. S28 and Tables S14–S17†).

**Fig. 2:**
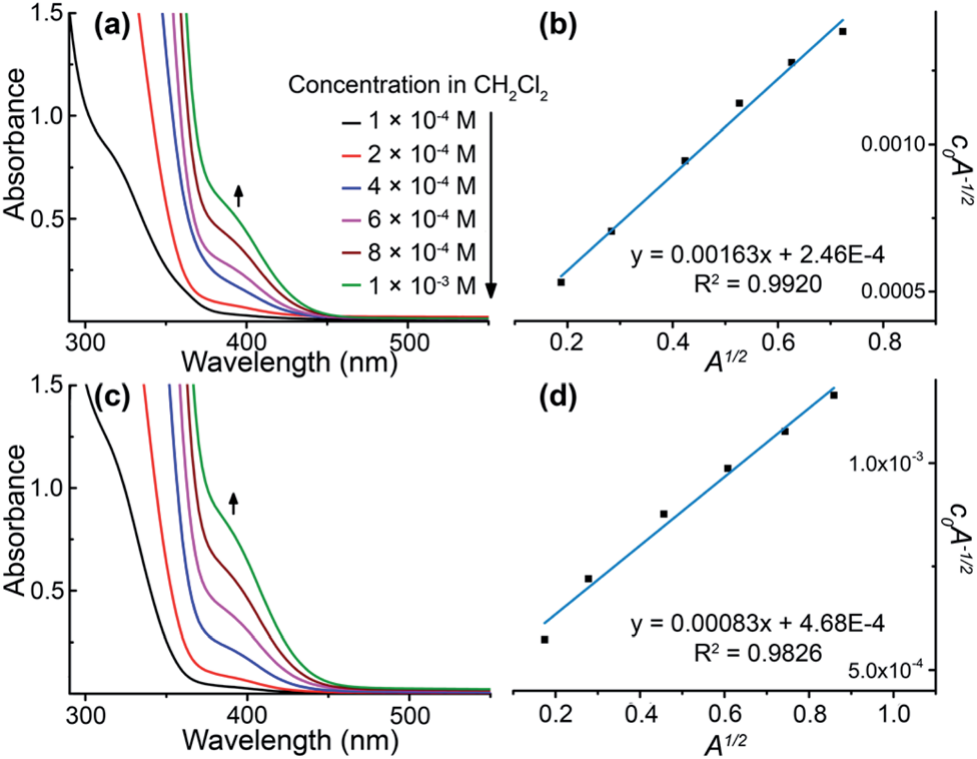
Varied-concentration UV-vis absorption spectra for (a) Au_6_Ag and (c) Au_9_Ag. Plots of *c*_0_*A*^−1/2^*versus A*^1/2^ taken at the respective low-energy absorption bands (392 and 394 nm), characteristic of dimers of (b) Au_6_Ag and (d) Au_9_Ag.

**Chart 1:**
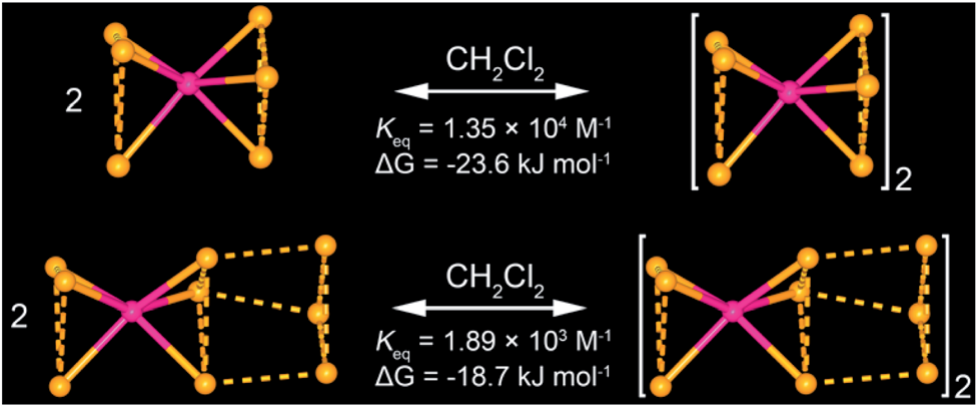
Illustrations of monomer–dimer aggregation in CH_2_Cl_2_ solution of (top) Au_6_Ag from heptanuclear to tetradecanuclear clusters and (bottom) Au_9_Ag from decanuclear to icosanuclear clusters. Only gold and silver atoms, and one set of disordered atoms, are presented for clarity purposes.

All of these homometallic and heterometallic clusters show good solubility in dichloromethane solution and are still strongly emissive. Unlike common phosphorescent complexes/clusters,^55–57^ it is exciting that these NCs are insensitive to oxygen quenching due to ligand-protected metal cores (Table S8†). Au_3_ exhibits a high-energy absorption peak at 264 nm and two distinct emission peaks at around 350 and 710 nm in CH_2_Cl_2_ solution (Fig. S22†), which fall into the near UV and infrared regions. As shown in Fig. 2a and c, both Au_6_Ag and Au_9_Ag exhibit a high-energy monomer absorbance peak at around 315 nm and an additional low-energy oligomer band at around 390 nm at higher concentrations. By calculations of apparent molar extinction coefficients, deviation from Beer's law is observed for the lower-energy absorption bands around 390 nm, as depicted and analysed in Fig. 2b, d, and S21,† respectively.^58^ Further analyses and good linearities confirm the assumption of monomer–dimer aggregations of both Au_6_Ag and Au_9_Ag in CH_2_Cl_2_ solution, giving rise to remarkable nanoscale clustering into an [Au_6_Ag]_2_ tetradecanuclear 14-metal-atom cluster and an [Au_9_Ag]_2_ icosanuclear 20-metal-atom cluster—giving rise to respective equilibrium constants (*K*_eq_) of *ca.* 1.35 × 10^4^ M^−1^ and 1.89 × 10^3^ M^−1^, and Gibbs free energy (Δ*G*) of −23.6 and −18.7 kJ mol^−1^ at 298 K (Table S6† and Chart 1).

**Fig. 3:**
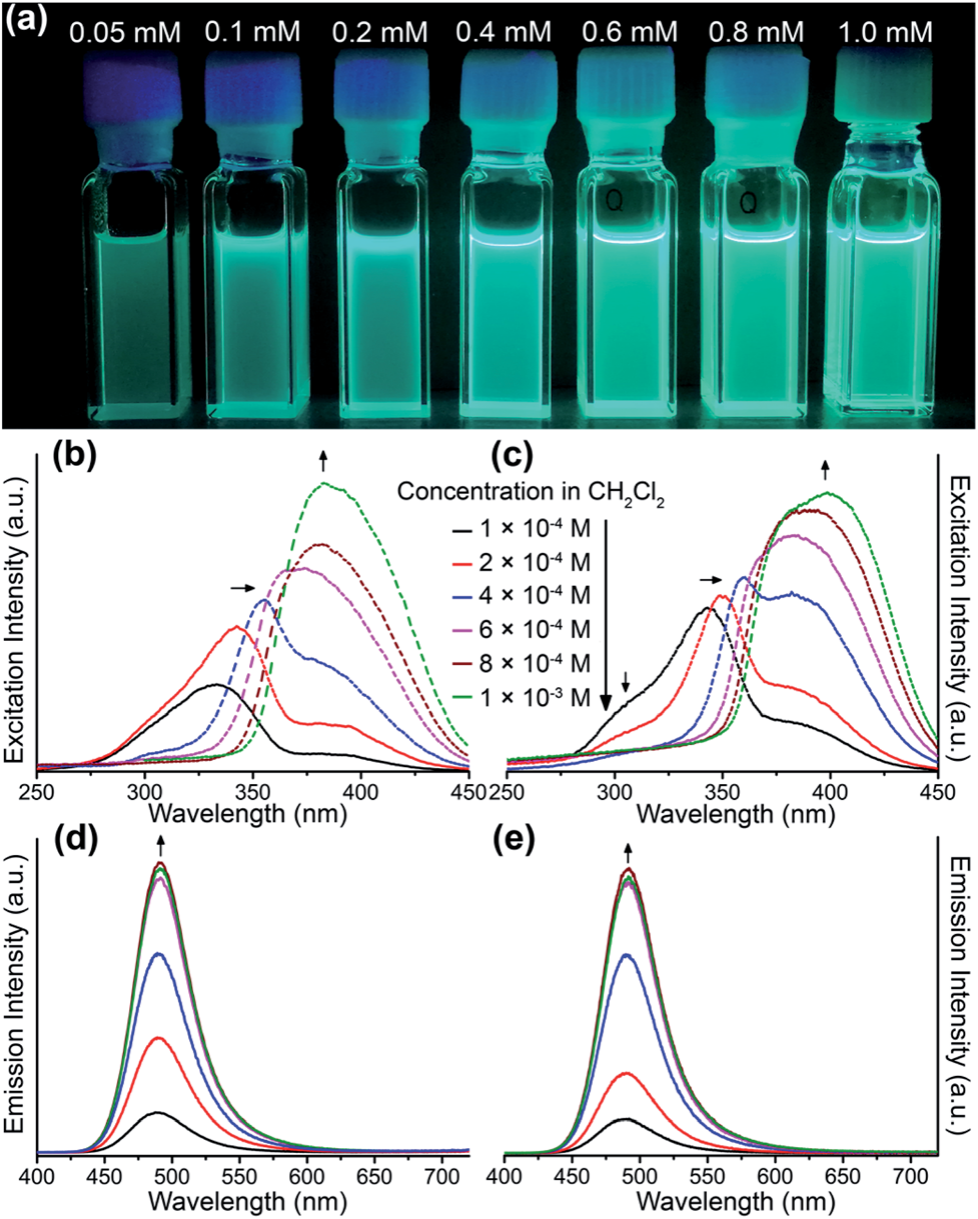
(a) Digital photographs of Au_6_Ag in CH_2_Cl_2_ solution of varied concentrations under 365 nm UV light. Varied-concentration excitation and emission spectra for (left: b and d) Au_6_Ag and (right: c and e) Au_9_Ag in CH_2_Cl_2_ solution under room temperature, respectively. The excitation wavelengths were 300 nm and the monitored emission wavelengths were 490 nm.

Solution-state photoluminescence measurements were also conducted for both heterobimetallic clusters and only Au_6_Ag shows a dependent correlation between the luminescence efficiency and concentration (Fig. 3a). Similar to the absorption spectra, a consistent trend of excitation energy *versus* concentration is also observed for both Au_6_Ag and Au_9_Ag (Fig. 3b and c), showing intensity increment of the low-energy band and bathochromic shift of the high-energy band, which finally combine into a broad excitation band at high concentrations.^45^ Moreover, the positive correlations between the luminescence intensity and concentrations of these two clusters are also verified. Different from the absorption and excitation, no spectral shift, but increased emission intensities, are observed when concentrating the gold–silver clusters in solution, both showing maxima at 491 nm (Fig. 3d and e), almost identical to that of solid-state emissions.

**Fig. 4:**
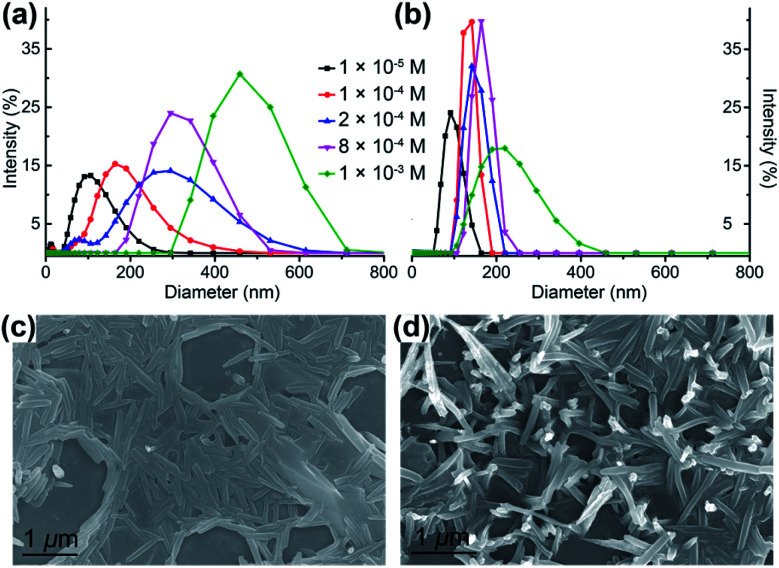
Dynamic light scattering (DLS) results for (a) Au_6_Ag and (b) Au_9_Ag characterizing the size distributions of the nanoaggregates in CH_2_Cl_2_ solution of different concentrations. Scanning electron microscope (SEM) images prepared from the 1.0 mM CH_2_Cl_2_ solution of (c) Au_6_Ag and (d) Au_9_Ag.

A further step is to demonstrate that the gold–silver clusters have indeed undergone aggregation processes in solution and to quantify the emission enhancement, *i.e.* fully confirming the AIEE phenomenon. Dynamic light scattering (DLS) experiments clearly reveal the generation of nanoaggregates of Au_6_Ag and Au_9_Ag (Fig. 4a, b and Table 1), which could also be witnessed by scanning electron microscopy (Fig. 4c, d and S10†). The average particle diameter of Au_6_Ag expands from 109 nm at 0.01 mM to 471 nm at 1.0 mM, while that of Au_9_Ag remains at around 150 nm in the range of 0.1 mM and 1.0 mM. As shown in Table 1, the size of the nanoaggregates directly influences the *Φ*_PL_ magnitudes, which range from *Φ*_PL_ of 27.0% to 60.6% for an average diameter of 179 nm to 471 nm, respectively, for Au_6_Ag. In contrast, Au_9_Ag exhibits steady *Φ*_PL_ values of around 37% and particle diameters of around 150 nm. It is interesting to observe such an unusual stoichiometric-ratio-dependent AIEE behaviour in a system of atomically precise clusters.

**Table 1:** Correlation of the concentrations of the clusters, sizes of the nanoaggregates and absolute *Φ*_PL_ for Au_6_Ag and Au_9_Ag in CH_2_Cl_2_ solution

Cluster	Concentration [mM]	Average particle diameter [nm]	*Φ*_PL_ [%]
Au_6_Ag	0.1	179	27.0
	0.2	283	31.6
	0.4	—	42.8
	0.6	—	51.8
	0.8	316	50.2
	1.0	471	60.6
Au_9_Ag	0.1	134	34.0
	0.2	148	36.4
	0.4	—	40.9
	0.6	—	36.4
	0.8	166	39.5
	1.0	216	34.2

A helpful insight to justify the *Φ*_PL_/AIEE trend variation in the hepta- *vs.* deca-nuclear clusters herein is provided by the calculated radiative/non-radiative decay rate constants (Au_6_Ag: 1.69/4.59 × 10^4^ s^−1^ at 0.1 mM, 3.19/2.07 × 10^4^ s^−1^ at 1.0 mM; Au_9_Ag: 2.05/3.98 × 10^4^ s^−1^ at 0.1 mM, 1.94/3.74 × 10^4^ s^−1^ at 1.0 mM). For Au_6_Ag, the inhibition of non-radiative decay upon aggregation is in line with the phenomenal description of AIE,^32^ but the promotion of the radiative transition efficiency is unconventional. Comparing the crystallographic disorder in the columnar structures of Au_6_Ag and Au_9_Ag (Fig. S14 and S15†), one might notice that the silver cations in the former experience partial occupancy disorder along the *C*_3_ axis. In the literature, weakly-disordered systems with reduced effective mass were suggested to have strong electron/hole mobility that is responsible for the enhanced photoluminescence.^20,59^ Besides, *via* quantitative analysis of solution-state absorption spectra, we have attained a 7× larger equilibrium constant (*K*_eq_) for Au_6_Ag than for Au_9_Ag, suggesting a stronger tendency for the former to aggregate in solution (Table S6† and Chart 1).

## Conclusions

Herein, different narratives for ligand-unsupported d^10^–d^10^ M–M′ interactions (with covalent bonding strength) and aggregation-induced emission enhancement (with larger than doubled *Φ*_PL_) are provided for sandwich-like gold–silver clusters through a combined experimental/computational study. The synergy effect, from Lewis acid/π-base interactions and metal–metal interactions (metallophilicity), gives rise to enhanced stability of the sandwich-like structures with ligand-unsupported Au–Ag bonding interactions and the comprehension of the bonding nature between d^10^ metals. The regulation of the stoichiometric ratio of Au : Ag in the nanoclusters with similar morphology results in nanocluster aggregation and further emission enhancement. Thus, a bridge is built between the effect of doping a foreign silver ion into cyclic trinuclear Au^I^ complexes and the photophysical properties regulated by the Au : Ag ratio in the aggregated nanoclusters.

## Experimental section

### Materials

All starting materials were purchased from commercial sources and used as received without further purification. The solvents used for synthesis were of analytical grade and those for the photophysical studies were of HPLC grade. Detailed characterization methods are included in the ESI.†

### Synthesis of *cyclo*-trimer gold(i) bis-3,5-(ethoxycarbonyl)_2_-pyrazolate Au_3_

To 15 mL of an ethanol solution of bis-3,5-(ethoxycarbonyl)_2_-1*H*-pyrazole (*H*L, 0.106 g, 0.5 mmol), 30 mL of an acetone solution of gold(tetrahydrothiophene)chloride (0.160 g, 0.5 mmol) was added. After the addition of a few drops of anhydrous triethylamine, a white precipitate immediately formed and the suspension was stirred for another 15 min to react completely. The white precipitate was collected by filtration and washed with methanol, acetone, and diethyl ether (3 × 1 mL for each) in a high yield (0.165 g, yield 82%) showing bright red emission under UV light. UV-vis in CH_2_Cl_2_ (*λ*_max_/nm): 264. FT-IR (KBr pellet, *ν*/cm^−1^): 3169w, 2984w, 1735s, 1465m, 1433m, 1387m, 1370m, 1257s, 1180s, 1089m, 1039m, 1018w, 848s, 759m, 628w. Elemental analyses for Au_3_C_27_O_12_N_6_H_33_, found: C, 26.82; H, 2.929; N, 6.75; calcd: C, 26.48; H, 2.716; N, 6.86. ^1^H-NMR (CDCl_3_, 400 MHz): *δ*/ppm = 7.60 (s, 2.7, –pyrazolate), 4.45 (q, 12.0, –CH_2_–), 1.40 (t, 18.5, –CH_3_).

### Synthesis of [Au_3_–Ag–Au_3_][PF_6_] (Au_6_Ag)

A mixture of AgPF_6_ (0.008 g, 0.03 mmol) and two equivalents of Au_3_ (0.077 g, 0.06 mmol) in 10 mL of CH_2_Cl_2_ solution was stirred under room temperature for 30 min to afford a greenish suspension. Needle-like crystals of [Au_3_–Ag–Au_3_][PF_6_] (denoted as Au_6_Ag) were obtained by diffusing *n*-hexane/diethylether (3 : 2, v/v) into the greenish transparent filtrate (0.068 g, yield 80%). MALDI-TOF-MS (α-cyano-4-hydroxycinnamic acid as the matrix): *m*/*z* for [Au_3_L_3_Ag]^+^: found 1333.060, calcd 1333.020; [Au_6_L_6_Ag]^+^: found 2557.198, calcd 2557.134. FT-IR (KBr pellet, *ν*/cm^−1^): 3169w, 2984m, 1128s, 1710s, 1531m, 1470m, 1428w, 1389w, 1369m, 1308m, 1271s, 1255s, 1230s, 1178s, 1080s, 1038m, 1018w, 852m, 762s, 628w. Elemental analyses for Au_6_C_54_O_24_N_12_H_66_AgPF_6_, found: C, 24.33; H, 2.305; N, 6.35. Calcd: C, 24.01; H, 2.462; N, 6.22. ^1^H-NMR (CDCl_3_ + CH_2_Cl_2_, 500 MHz): *δ*/ppm = 6.68 (br, 6.2, –pyrazolate), 4.04 (br, 24.0, –CH_2_–), 1.21 (br, 36.8, –CH_3_).

### Synthesis of {[Au_3_]_2_–Ag–Au_3_}[PF_6_] (Au_9_Ag)

A mixture of AgPF_6_ (0.008 g, 0.03 mmol) and more than three equivalents of Au_3_ (0.123 g, 0.10 mmol) in 10 mL of CH_2_Cl_2_ solution was stirred under room temperature for 30 min to afford a greenish suspension. After evaporating off the greenish filtrate under room temperature, block-shaped crystals along with a black solid could be found in the vial. A mixed solvent of ethyl acetate and CH_2_Cl_2_ (1 : 10, v/v) was used to dissolve the crystals and insoluble substances were filtered out. Block-shaped crystals of {[Au_3_]_2_–Ag–Au_3_}[PF_6_] (denoted as Au_9_Ag) were obtained by evaporating the greenish transparent ethyl acetate/CH_2_Cl_2_ filtrate under room temperature (0.056 g, yield 47%). MALDI-TOF-MS (α-cyano-4-hydroxycinnamic acid as the matrix): *m*/*z* for [Au_3_L_3_Ag]^+^, found 1333.009, calcd 1333.020; [Au_4_L_4_Ag]^+^, found 1741.057, calcd 1741.059; [Au_6_L_6_Ag]^+^: found 2557.138, calcd 2557.134. FT-IR (KBr pellet, *ν*/cm^−1^): 2982m, 1740s, 1716s, 1635w, 1526w, 1472m, 1435m, 1386m, 1368m, 1254s, 1174s, 1082m, 1038m, 843s, 757m, 626w, 558w. Elemental analyses for Au_9_C_81_O_36_N_18_H_99_AgPF_6_, found: C, 24.96; H, 2.578; N, 6.53. Calcd: C, 24.78; H, 2.541; N, 6.42. ^1^H-NMR (CDCl_3_ + CH_2_Cl_2_, 500 MHz): *δ*/ppm = 7.44 (br, 10.9, –pyrazolate), 4.40 (br, 36.0, –CH_2_–), 1.39 (t, 52.4, –CH_3_).

### Crystallographic study

X-ray crystallographic data were collected on a XtaLab PRO MM007HF DW Diffractometer System equipped with a MicroMax-007DW MicroFocus X-ray generator and Pilatus 200K silicon disarray detector (Rigaku, Japan, Cu Kα, *λ* = 1.54184 Å or Mo Kα, *λ* = 0.71073 Å) under 293 K or 100 K. Data reductions were performed on CrysAlisPro. Structures were solved by using direct methods by ShelXT for Au_3_ and SIR2004 for Au_9_Ag in the OLEX2 program package, and all non-hydrogen atoms were refined anisotropically by the full-matrix least-squares method on *F*^2^ by using the ShelXL program.^60^ The hydrogen atoms were located from different maps and refined with isotropic temperature factors. In the Au_9_Ag structure, Au and Ag atoms exhibit positional disorder. Detailed structure refined information is appended in the CIF file.

For Au_6_Ag, a fully-satisfactory crystal structure of the sample could not be obtained *via* single-crystal XRD (SCXRD) directly due to the single crystal batch's weak diffraction and the disorder problem of the ligands. The cell parameters and heavy atomic positions (Au and Ag) were generated from SCXRD data *via* the Patterson method; ligands were built based on the structure of Au_3_ and refined by Rietveld refinement in the Reflex module of Materials Studio (residuals: *R*_p_ = 12.65%, *R*_wp_ = 19.86%). The results of EDX, mass spectra, and elemental analyses could fit the model very well. The Ag atoms in Au_6_Ag are partially occupied along the *c* axis in the *P*6̄ space group and, therefore, the repeating unit [along *c*] consists of alternating {3 Au_6_Ag, 1 Au_3_ and 1 Au_9_Ag_2_}/{1 Au_9_Ag_2_ 1 Au_3_ and 3 Au_6_Ag} clusters (Fig. S13 and S14†). The silver atoms in the Au_9_Ag_2_ cluster adopt a face-sharing octahedral geometry Au_3_–Ag-(μ-Au)_3_–Ag–Au_3_. Based on the refinement results, silver atoms except Ag1 are half-occupied and the Au–Ag distances range from 2.641(7) to 2.713(6) Å. The DFT-computed Au–Ag distances and their reasonable agreement with the non-disordered experiment in the Au_9_Ag model should lend some credibility to the DFT-computed Au–Ag distances in the Au_6_Ag model, which only showed reasonable distances in the 2.7 Å range.

Crystal data and structure refinement parameters are summarized in Table S1.† Selected bond lengths and angles are given in Tables S2 – S4.† CCDC no. 1968678 and 1968679 for Au_3_ and Au_9_Ag.

## Conflicts of interest

There are no conflicts to declare.

## Supplementary Material

SC-012-D0SC05095D-s001

SC-012-D0SC05095D-s002
