# Correction: Aggregation-induced phosphorescence sensitization in two heptanuclear and decanuclear gold–silver sandwich clusters

**DOI:** 10.1039/d0sc90280b

**Published:** 2020-12-23

**Authors:** Zhou Lu, Yu-Jie Yang, Wen-Xiu Ni, Mian Li, Yifang Zhao, Yong-Liang Huang, Dong Luo, Xiaoping Wang, Mohammad A. Omary, Dan Li

**Affiliations:** College of Chemistry and Materials Science, Guangdong Provincial Key Laboratory of Functional Supramolecular Coordination Materials and Applications, Jinan University Guangzhou 510632 P. R. China danli@jnu.edu.cn; Department of Chemistry, University of North Texas 1155 Union Circle #305070 Denton Texas 76203 USA omary@unt.edu; Department of Chemistry, Shantou University Guangdong 515063 P. R. China; Department of Chemistry, Shantou University Medical College Shantou Guangdong 515041 P. R. China; Neutron Scattering Division, Oak Ridge National Laboratory Oak Ridge Tennessee 37831-6475 USA

## Abstract

Correction for ‘Aggregation-induced phosphorescence sensitization in two heptanuclear and decanuclear gold–silver sandwich clusters’ by Zhou Lu *et al.*, *Chem. Sci.*, 2020, DOI: 10.1039/d0sc05095d.

In the original manuscript, an acknowledgement to the National Science Foundation and the University of North Texas should have been included. A corrected Acknowledgements section is detailed below:

This work was financially supported by the National Natural Science Foundation of China (No. 21731002, 21975104, and 21801095), the Guangdong Major Project of Basic and Applied Research (2019B030302009), the China Postdoctoral Science Foundation (2017M622894) and Jinan University. MAO acknowledges supporting aspects of his group’s contributions by the Welch Foundation (Grant B-1542), U.S. National Science Foundation (CHE-1413641), and the Shenzhen/China Peacock Plan (No. 1208040050847074). We appreciate Dr Xue Li and Xue Zhi Wang (JNU) for the help with the mass spectrometry studies, and Dr Vladimir Nesterov (UNT) for the help with the crystallography studies. The authors also acknowledge the National Science Foundation’s MRI Program (CHE-1726652) and the University of North Texas for supporting the acquisition of the Rigaku XtaLAB Synergy-S X-ray diffractometer, which was used to aid the troubleshooting of a disordered structure by re-acquiring an independent data set and refining it.

The Royal Society of Chemistry apologises for these errors and any consequent inconvenience to authors and readers.

## Supplementary Material

