# Supplementary material for: Aggregation-induced phosphorescence sensitization in two heptanuclear and decanuclear gold–silver sandwich clusters
Source: Chem Sci. 2020 Oct 30;12(2):702–8. doi: 10.1039/d0sc05095d (PMC8179010; doi:10.1039/d0sc05095d)
Supplement: SC-012-D0SC05095D-s001 [file SC-012-D0SC05095D-s001.pdf]

Electronic Supplementary Material (ESI) for Chemical Science.
This journal is © The Royal Society of Chemistry 2020

Electronic Supplementary Information for

Aggregation-Induced Phosphorescence Sensitization in Two Heptanuclear and Decanuclear Gold-Silver Sandwich Clusters

Zhou Lu,†[ab] Yu-Jie Yang,†[c] Wen-Xiu Ni,[d] Mian Li,[c] Yifang Zhao,[a] Yong-Liang Huang,[a] Dong Luo,[a] Xiaoping Wang,[e] Mohammad A. Omary,*[b] and Dan Li*[a]

[a.] College of Chemistry and Materials Science, Guangdong Provincial Key Laboratory of Functional Supramolecular Coordination Materials and Applications, Jinan University, Guangzhou 510632, P. R. China. Email: danli@jnu.edu.cn

[b.] Department of Chemistry, University of North Texas, 1155 Union Circle #305070, Denton, Texas 76203, United States. Email: omary@unt.edu

[c.] Department of Chemistry, Shantou University, Guangdong 515063, P. R. China.

[d.] Department of Chemistry, Shantou University Medical College, Shantou, Guangdong 515041, P. R. China.

[e.] Neutron Scattering Division, Oak Ridge National Laboratory, Oak Ridge, Tennessee 37831-6475, United States.

†. Z.L. and Y.-J.Y contributed to this work equally.

Experimental Section

Materials and Physical Measurements

All starting materials were purchased from commercial sources and used as received without further purification. Fourier-transform infrared (FT-IR) spectra were measured using a Nicolet Avatar 360 FT-IR spectrophotometer. Thermogravimetric analysis (TGA) was carried out in a nitrogen stream using TA Instruments Q50 TGA thermal analysis equipment with a heating rate of 10 °C min^{-1}. Solution UV-vis absorption spectra were recorded on an Agilent UV-vis spectrometer 8453 and a Perkin-Elmer Lambda 900 UV/Vis/NIR spectrometer; solid-state UV-Vis absorption spectra were recorded on a Bio-Logic MOS-500 multifunctional circular dichroism spectrometer. Mass spectra were obtained on an Applied Biosystems 4800 Plus MALDI TOF Analyzer (ABI) spectrometer using α-Cyano-4-hydroxycinnamic acid (CHCA) as matrix. Elemental analyses were performed by Elementar vario EL Cube equipment. X-ray photoelectron spectroscopies (XPS) were obtained Thermo Fisher Scientific ESCALAB 250Xi (monochrome Al Kα hν = 1486.6 eV, power = 150 W, beam spot = 500 μm, binding energy C1s = 284.8 eV). The dynamic light scattering (DLS) measurements were performed on Malvern Zetasizer Nano-ZS. Energy-dispersive X-ray spectroscopy (EDX) was performed on a scanning electron microscopy (SEM, Zeiss Gemini 300 Field Emission Scanning Electron Microscope) equipped with energy dispersive X-ray spectrometer operated at 15 kV. Powder X-ray diffraction (PXRD) experiments were performed on a Rigaku Ultima IV X-ray diffractometer (Cu Kα, λ = 1.5418 Å). NMR studies were conducted by Bruker Ascend 400 MHz and Varian Inova 500 MHz spectrometer and coaxial insert tubes for internal reference technique were adopted for **Au_6Ag** and **Au_9Ag**, where inside tube contained two drops of concentrated CH_2Cl_2 solution of heterobimetallic Au/Ag cluster and outside tube contained blank deuterated chloroform. The reason why we adopted this technique was that both deuterated dichloromethane solutions of heterometallic Au/Ag clusters turned into dark with insoluble black species in several minutes but CH_2Cl_2 solutions could keep in a good shape for a long time period (*vide infra*, Figure S18). The deuterated dichloromethane was purchased from J&K Scientific, *Ltd.* (D, 99.8 atom%, Cat. No.: 334919, 10×0.75ML), Cambridge Isotope Laboratories, *Inc.* (D, 99.8 atom%, item: DLM-23-5×1; D, 99.8 atom% + 0.05% v/v TMS, item: DLM-23TB-10×0.6), and Sigma-Aldrich, *Inc.* (D, 99.9 atom%, SKU: 444324-10×1ML) to conduct parallel experiments and avoid trace impurify. The study on stability of heterometallic Au/Ag clusters in contacting deuterated solvents is still ongoing.

Room-temperature steady-state photoluminescence spectra (PL) for all samples were recorded on a PTI QM/TM spectrofluorometer (Birmingham, NJ, USA). Corrections of excitation and emission for the detector response were performed ranging from 250-750 nm. Decay curves excited by a N_2 laser at 337 nm were recorded on the same instrument, and lifetimes were calculated using the FelixGX advanced photoluminescence phosphorescence software. Varied-temperature emission spectra were measured on an Edinburgh FLS920 spectrometer equipped with a continuous Xe900 Xenon lamp and a closed cycle cryostat (Advanced Research Systems). The absolute quantum yield was measured at room temperature by employing Hamamatsu C11347-11 absolute PL quantum yield spectrometer. The radiative rate constant is calculated based on the following formula k_r (s^{-1}) = Φ_{PL}/τ and the non-radiative decay rate follows k_{nr} (s^{-1}) = $(1-\Phi_{PL})/\tau$. In solid samples, crystalline samples were used for the photoluminescence measurements. The crystalline phase purity of the samples is assured by elemental analyses and powder X-ray diffraction measurements.

Characterizations

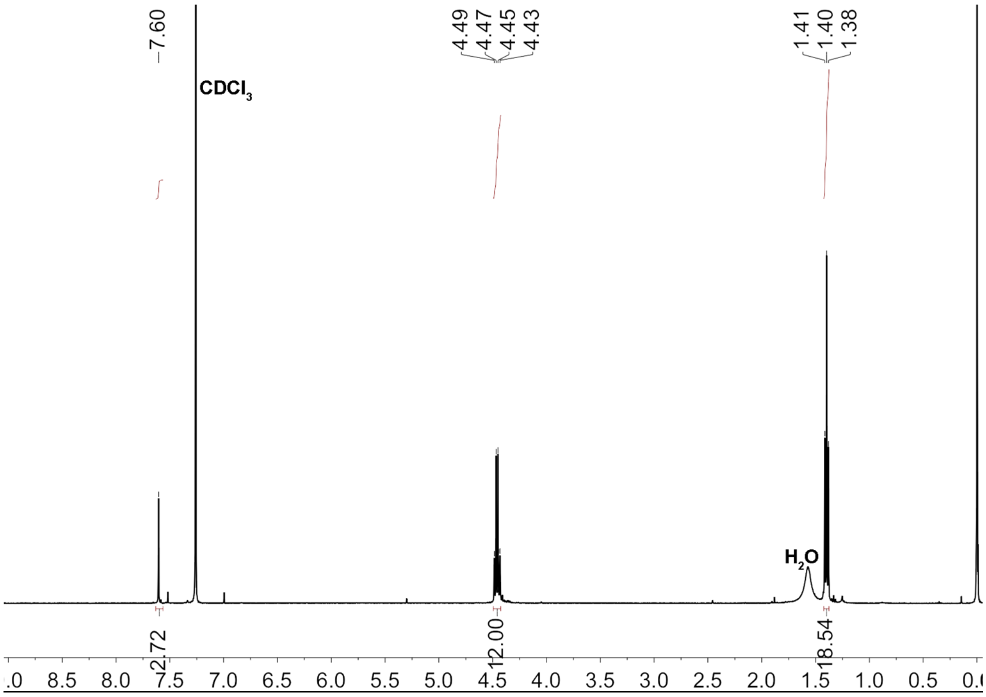

Figure S1. ^{1}H-NMR spectra of **Au$_3$** in d-chloroform.

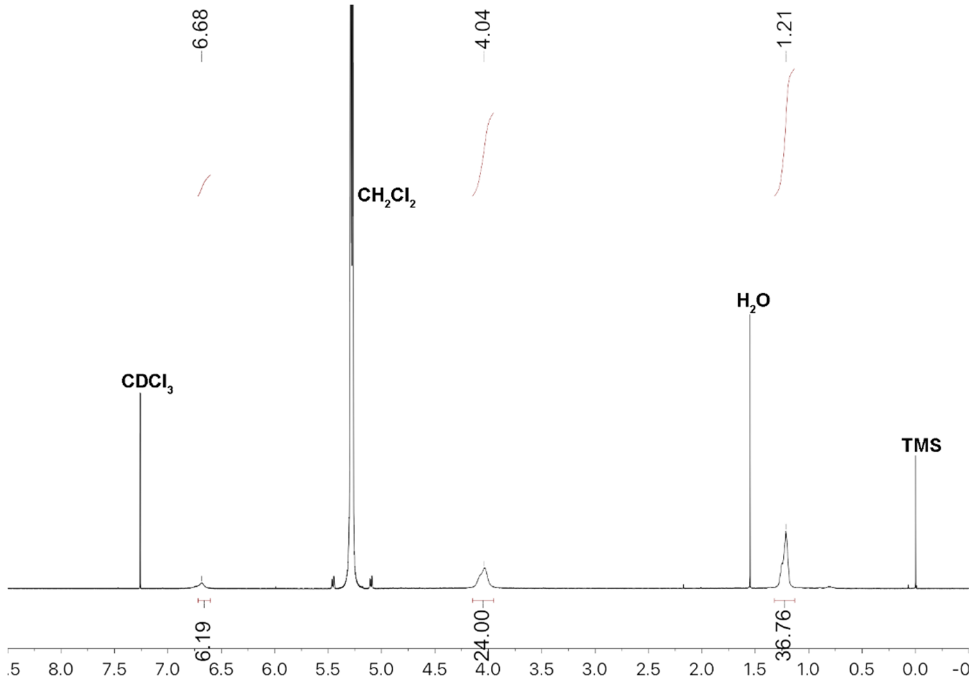

Figure S2. ^{1}H-NMR spectra of **Au$_6$Ag** in d-chloroform and dichloromethane.

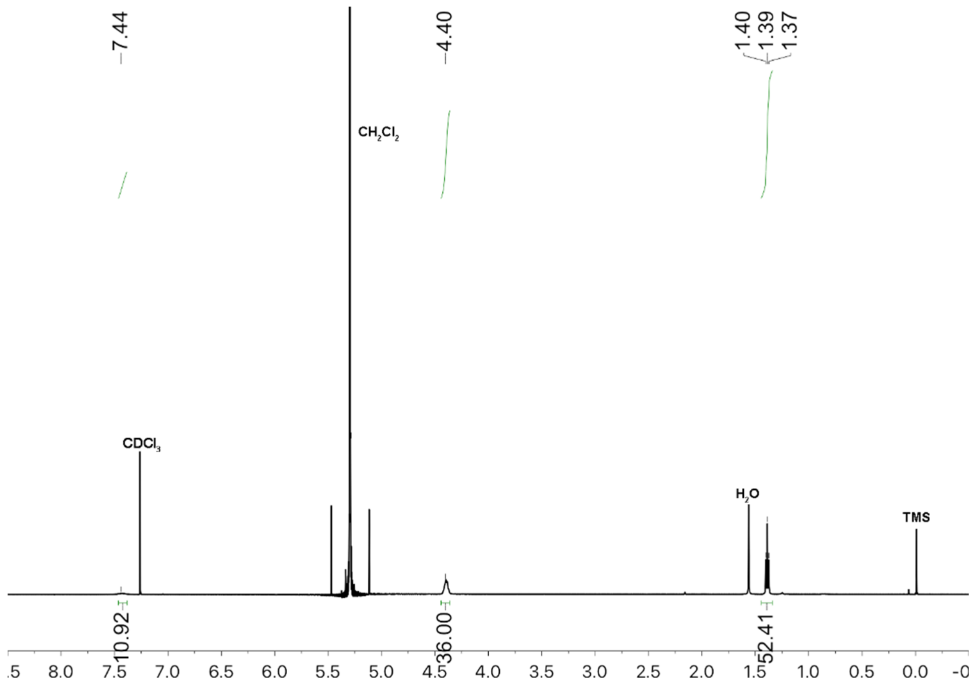

Figure S3. ^{1}H-NMR spectra of **Au9Ag** in d-chloroform and dichloromethane.

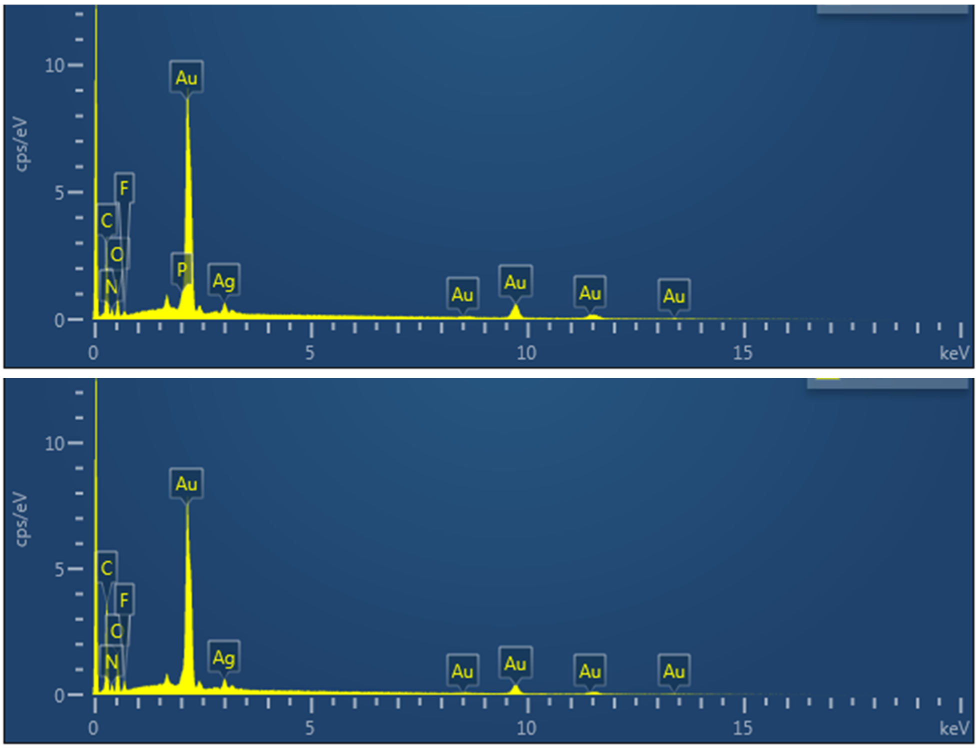

Figure S4. Energy-dispersive X-ray spectra (EDX) analysis of (top) **Au6Ag** and (bottom) **Au9Ag**. Calcd. for **Au6Ag** (%): Au, 85.7; Ag, 14.3; Found: Au, 86.3; Ag, 13.7; Calcd. for **Au9Ag** (%): Au, 90.0; Ag, 10.0; Found: Au, 89.9; Ag, 10.1.

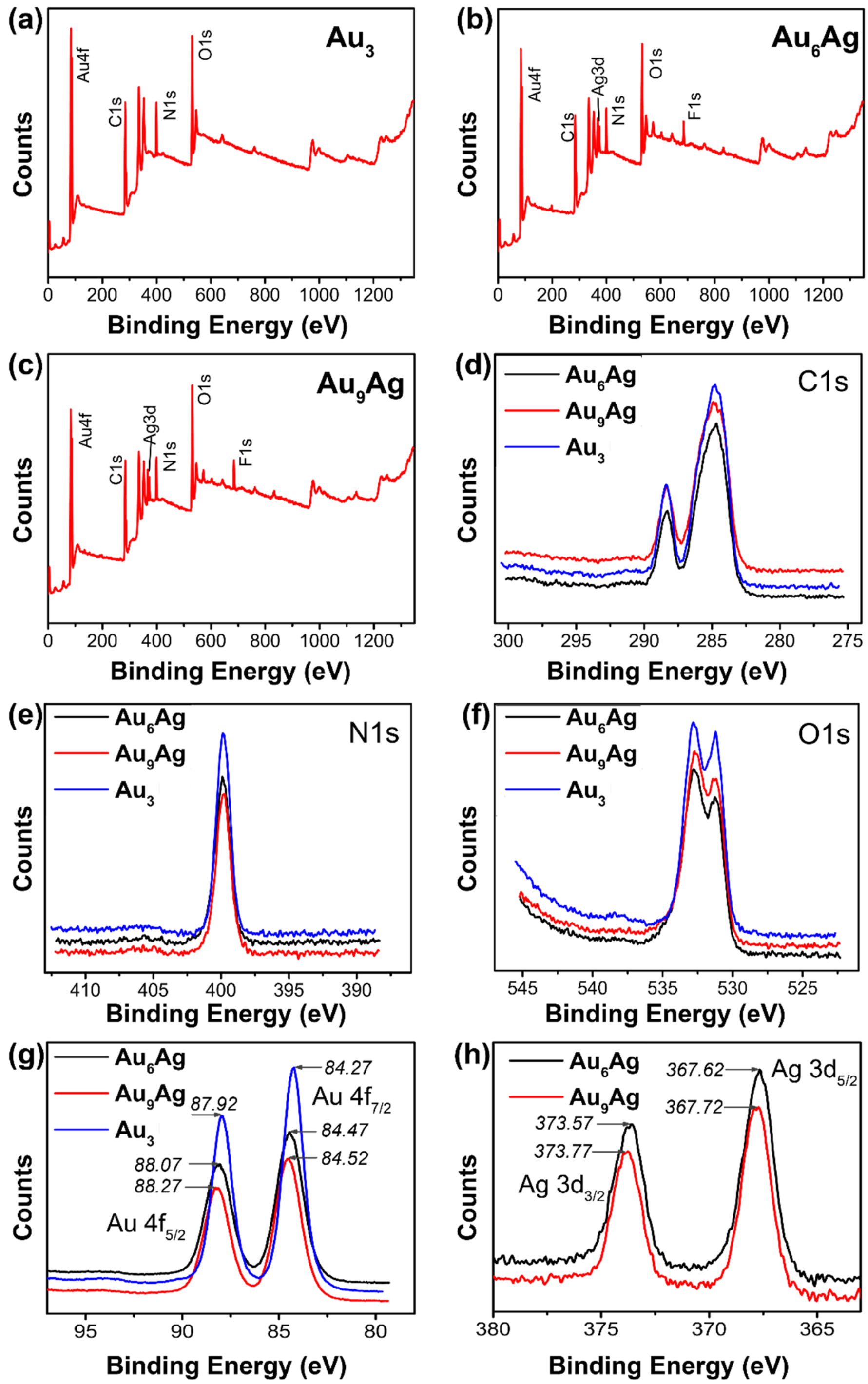

Figure S5. XPS spectra of (a) **Au$_3$**, (b) **Au$_6$Ag**, and (c) **Au$_9$Ag**. XPS binding energies of (d) C 1s, (e) N 1s, (f) O 1s, (g) Au 4f regions for **Au$_3$**, **Au$_6$Ag**, and **Au$_9$Ag**. XPS binding energies of (h) Ag 3d region for **Au$_6$Ag** and **Au$_9$Ag**.

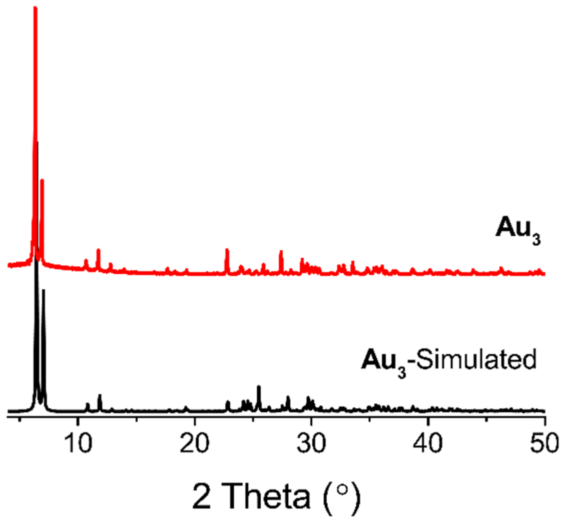

Figure S6. PXRD patterns for (black) simulated and (red) as-synthesized of **Au3**.

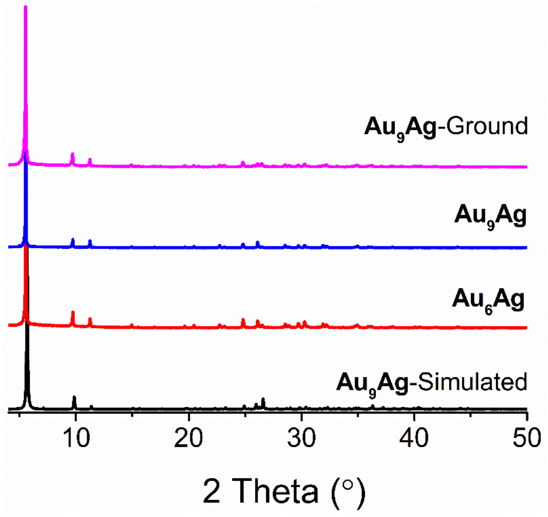

Figure S7. PXRD patterns for (black) simulated, (blue) as-synthesized and (magenta) ground sample of **Au9Ag** and for (red) as-synthesized of **Au6Ag**.

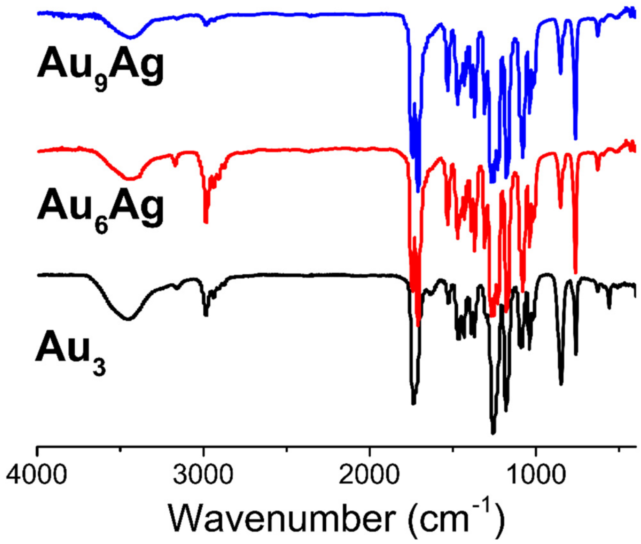

Figure S8. FT-IR spectra of **Au3, Au6Ag**, and **Au9Ag**.

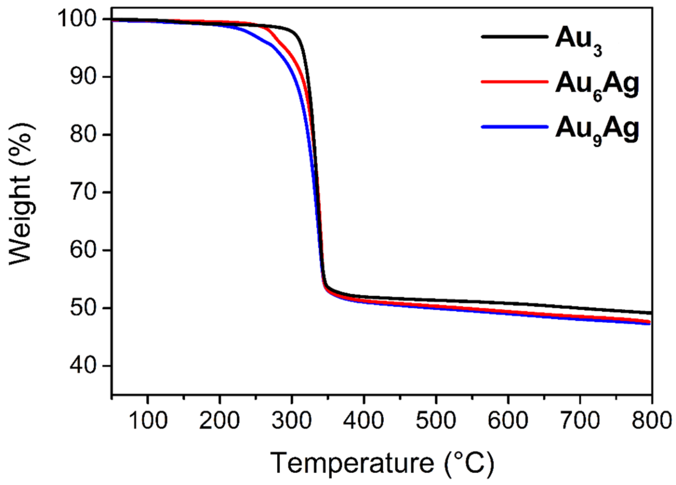

Figure S9. Thermogravimetric analysis (TGA) curves of **Au_3**, **Au_6Ag**, and **Au_9Ag**.

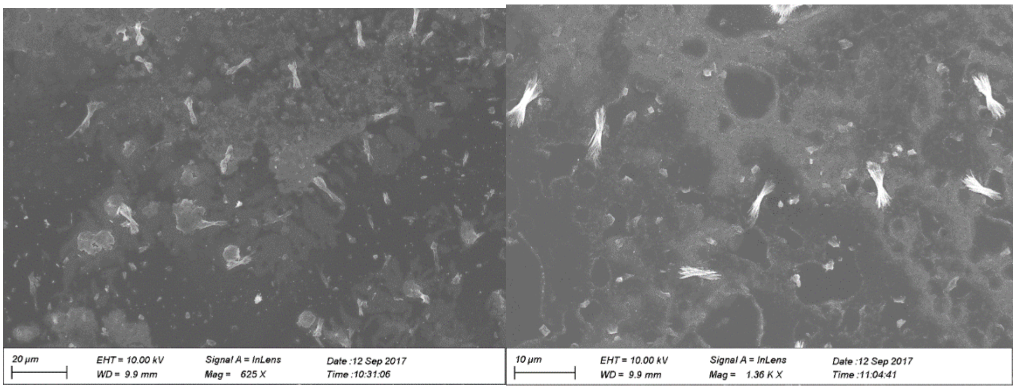

(a) (b)

Figure S10. Scanning electron microscope (SEM) images prepared from the 0.1 mM CH_2Cl_2 solution of (a) **Au_6Ag** and (b) **Au_9Ag**.

Crystallographic Study

Table S1. Summary of crystal data and structure refinement parameters for $\mathbf{Au_3}$, $\mathbf{Au_9Ag}$, and $\mathbf{Au_6Ag}$.

sample	$\mathbf{Au_3}$	$\mathbf{Au_9Ag}$	$\mathbf{Au_6Ag}$
Empirical formula	$Au_3C_{27}O_{12}N_6H_{33}$	$Au_9C_{81}O_{36}N_{18}H_{99}AgPF_6$	$Au_6C_{54}O_{24}N_{12}H_{66}AgPF_6$
CCDC No.	1968678	1968679	-
Formula weight	1224.49	3926.31	2701.81
Temperature (K)	293.00(10)	293(2)	100(2)
Crystal system	monoclinic	hexagonal	hexagonal
Space group	$P2_1/c$	$P\text{-}6_{2c}$	P-6
a (Å)	8.03090(10)	17.9776(13)	17.7387(4)
b (Å)	16.3497(2)	17.9776(13)	17.7387(4)
c (Å)	25.1896(3)	20.5845(14)	33.7730(10)
β (°)	94.2110(10)	-	-
Volume (Å^3)	3298.54(7)	5761.5(9)	9203.3(5)
Z	4	2	6
$D_{calcd.}$ (g/cm^3)	2.466	2.263	2.529
F (000)	2280.0	3652.0	6565.0
μ (mm^{-1})	25.203	11.681	24.813
2θ range for data collection (°)	6.45 - 148.302	7.202 - 52.736	5.234 - 147.598
reflns. collected	20445	28858	29799
Data / restraints / parameters	6535 / 0 / 439	4039 / 7 / 274	10873 / 5 / 70
R_{int}	0.0567	0.0321	0.0521
Goodness-of-fit on F^2	1.053	1.019	1.682
R_1 [I>2σ(I)] ^{a}, wR_2 [I>2σ(I)] ^{b}	0.0429, 0.1166	0.0219, 0.0481	0.1232, 0.3556
R_1 [all data] ^{a}, wR_2 [all data] ^{b}	0.0488, 0.1208	0.0311, 0.0511	0.1438, 0.3931

^{a} $R_1 = \sum|F_o| - |F_c||/\sum|F_o|$. ^{b} $wR_2 = \{[\sum w(F_o^2\text{-}F_c^2)^2]/\sum[w(F_o^2)^2]\}^{1/2}$; $w = 1/[\sigma^2(F_o^2) + (aP)^2 + bP]$, where $P = [\max(F_o^2,0) + 2F_c^2]/3$ for all data

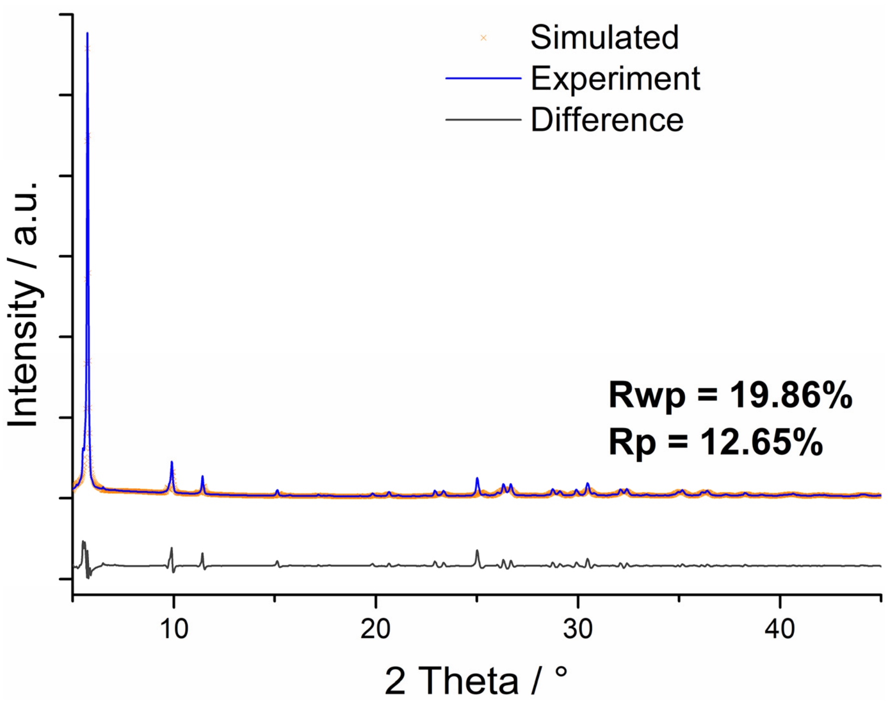

Figure S11. Rietveld refinement of PXRD patterns of **Au_6Ag** under 298 K.

Table S2. Comparison of selected bond length (Å) for **Au_3**, **Au_9Ag**, and **Au_6Ag**.

	**Au_3**	**Au_9Ag**#1	**Au_9Ag**#2	**Au_6Ag**
Au-N	2.012(6), 2.017(6), 2.026(6), 2.038(6), 2.039(6), 2.043(6)	2.011(10), 2.016(8), 2.033(9), 2.047(5)	2.002(8), 2.010(8), 2.012(8), 2.033(9)	- [a]
Au-Au (intra-trimer)	3.3883(5), 3.4008(6), 3.4180(5)	3.4186(8), 3.4886(14)	3.4186(8), 3.456(7)	3.424(5), 3.457(6), 3.538(7), 3.548(5), 3.574(6) 3.705(8), 3.705(19),
Au-Au (inter-trimer) [b]	3.3676(4), 3.5097(5)	3.2756(4)	3.175(6), 3.649(3)	3.210(2)
Au-Ag	-	2.7871(7)	2.617(5), 2.735(7)	2.641(7), 2.644(2), 2.647(2), 2.679(3), 2.679(6), 2.688(7), 2.691(3), 2.713(6)

[a] In **Au_6Ag**, the positions of ligand could not be determined by single-crystal X-ray crystallography due to disordering. [b] Only inter-trimer Au-Au distances shorter than 4.0 Å are under consideration.

Table S3. Selected bond angles (°) of **Au₃**.

N(1)-Au(1)-Au(2)#1	85.07(16)	N(6)-Au(1)-Au(2)#1	94.79(17)
N(6)-Au(1)-N(1)	179.7(3)	N(5)-Au(3)-N(4)	176.8(2)
N(2)-Au(2)-Au(1)#1	95.60(16)	N(2)-Au(2)-N(3)	176.6(2)
N(3)-Au(2)-Au(1)#1	87.81(17)		

Symmetry code: #1 2-x,1-y,1-z

Table S4. Selected bond angles (°) of **Au₉Ag**.

N(2)#2-Au(1)-N(1)	178.5(4)	N(2)-N(1)-Au(1)	118.3(8)
N(3)-Au(2Ab)-N(4)#2	164.8(3)	N(1)-N(2)-Au(1)#3	123.2(8)
N(4)-N(3)-Au(2)	121.9(6)	N(4)-N(3)-Au(2A)	120.6(6)
N(3)-N(4)-Au(2)#3	121.5(6)	N(3)-N(4)-Au(2A)#3	121.2(6)

Symmetry codes: #1 +y,+x,1-z; #2 1+y-x,2-x,+z; #3 2-y,1+x-y,+z; #4 +x,+y,1/2-z; #5 +y-x,1-x,+z; #6 1-y,1+x-y,+z; #7 +y-x,1-x,1/2-z; #8 1-y,1+x-y,1/2-z

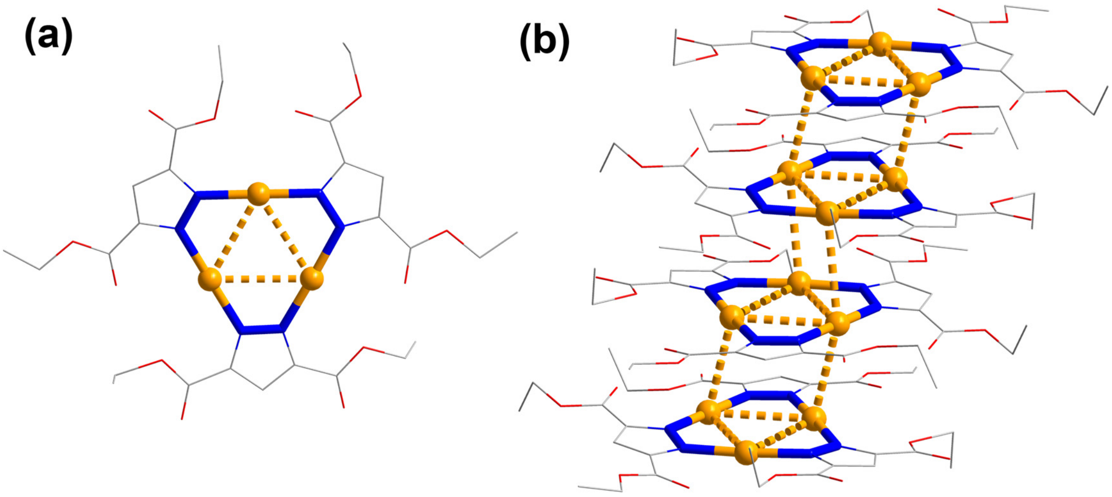

Figure S12. (a) Asymmetric unit of **Au₃** crystal structure at 293 K. (b) Packing diagram of **Au₃**. Colour representations: yellow, gold; blue, nitrogen; grey, carbon; red, oxygen. Hydrogen atoms are omitted from the figure for clarity.

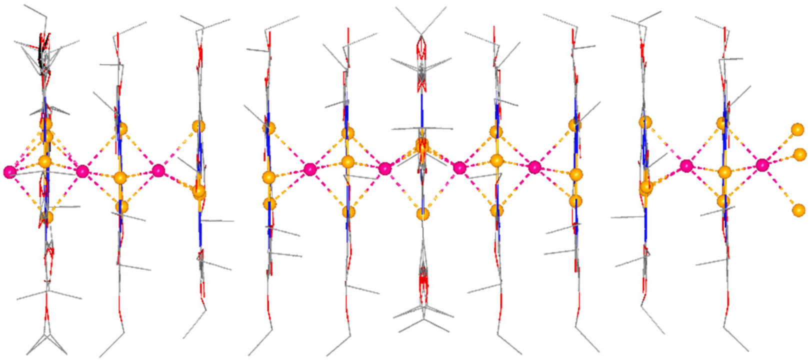

Figure S13. Packing diagram of **Au_6Ag** at 100 K. Note that all silver cations are partially occupied; three of four silver atoms are half occupied and the remaining one silver atom is fully occupied. Colour representations: yellow, gold; magenta, silver; blue, nitrogen; grey, carbon; red, oxygen. Hydrogen atoms and counter-anions are omitted from the figure for clarity.

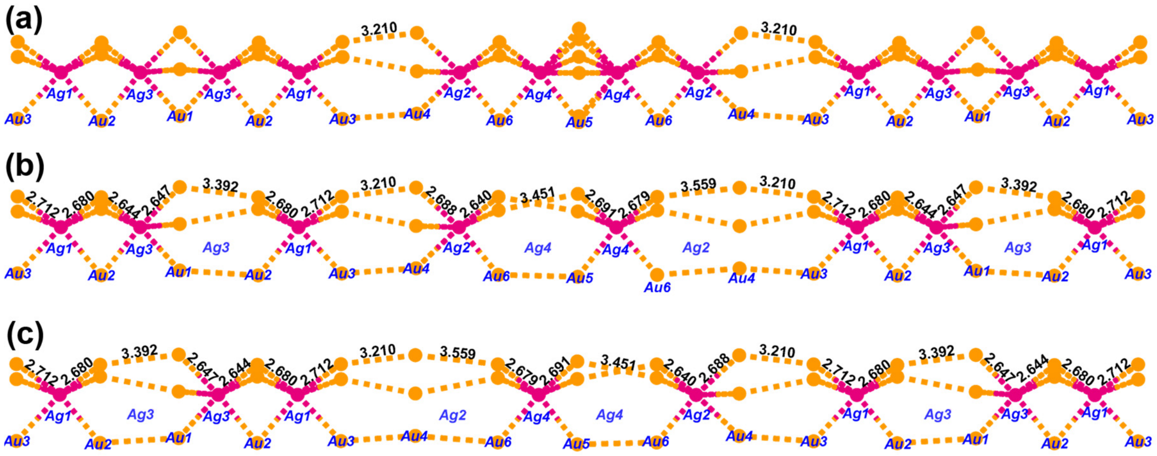

Figure S14. Distribution of Ag atoms in the gold-silver clusters of **Au_6Ag**. (a) Structure from X-ray diffraction. Silver atoms except Ag1 are half-occupied. (b, c) Local structures showing the two possible arrangements of silver atoms along the crystallographic *c*-direction with alternating {3 Au_6Ag, 1 Au_3 and 1 Au_9Ag_2} / {1 Au_9Ag_2, 1 Au_3 and 3 Au_6Ag} clusters as the repeating unit. The disordered Au atom (Au5B) and the ligands were omitted for clarity.

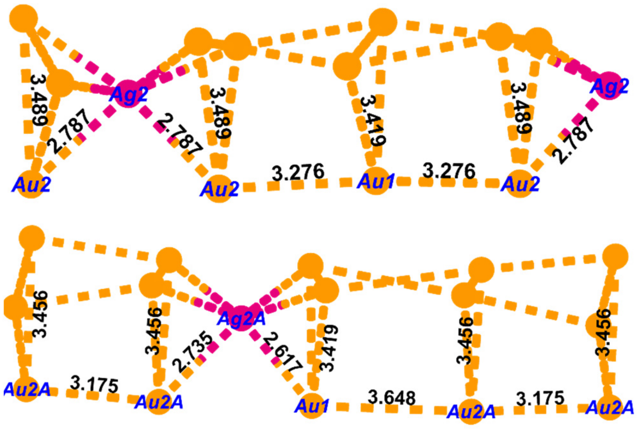

Figure S15. Illustrations of two different disordering sets of **Au_9Ag** (Colour representations: yellow, gold; magenta, silver. Ligands and counter-anions are omitted from the figures for clarity).

Photoluminescence Measurement

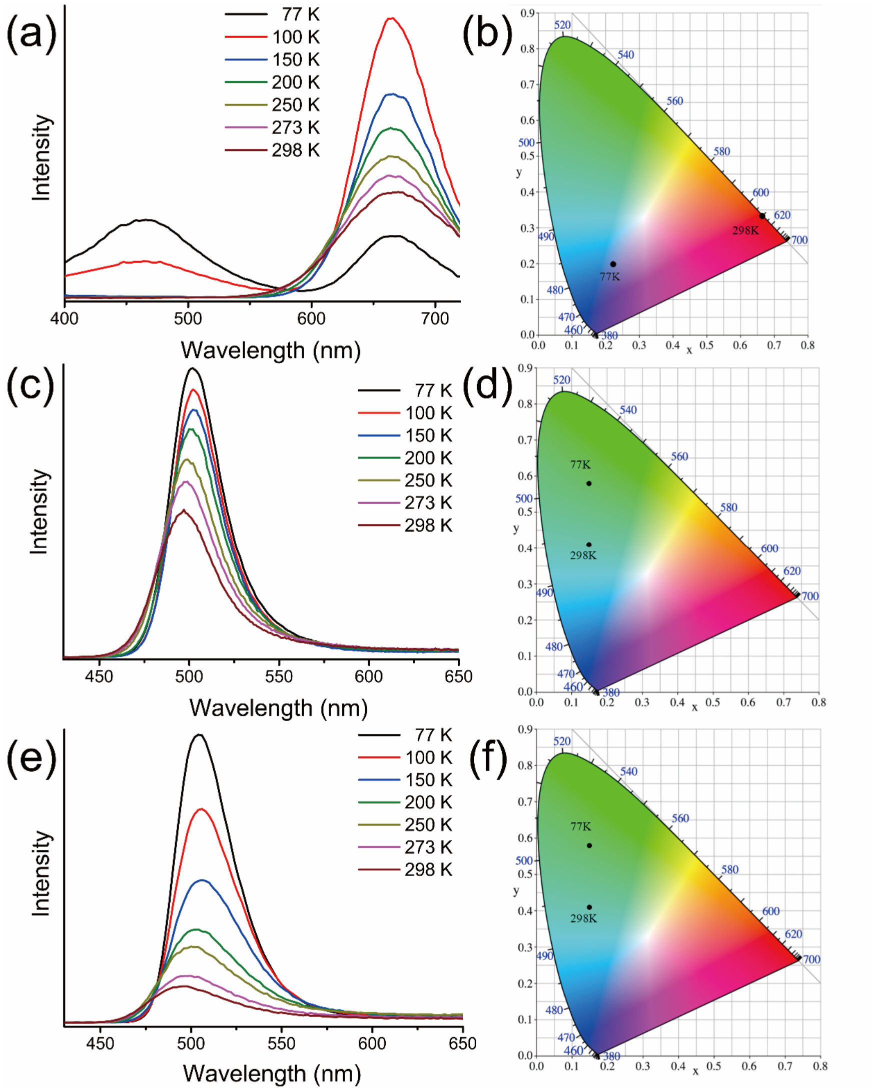

Figure S16. Varied-temperature solid-state emission spectra of (a) **Au_3** (Ex = 300 nm), (c) **Au_6Ag** (Ex = 360 nm), and (e) **Au_9Ag** (Ex = 360 nm). Emission colour profiles of (b) **Au_3**, (d) **Au_6Ag**, and (f) **Au_9Ag** in CIE-1931 chromaticity diagram at 298 K and 77 K.

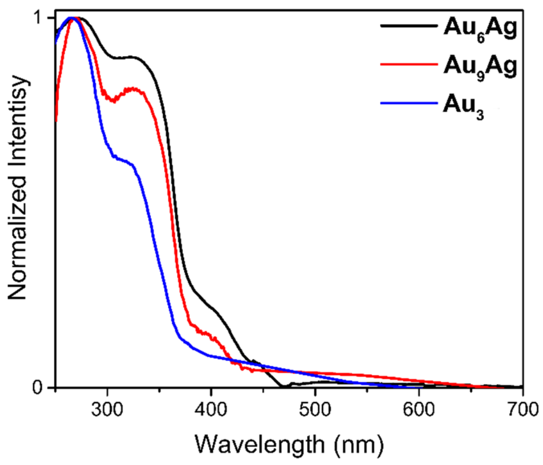

Figure S17. Solid state UV-vis spectra of three nanoclusters.

Table S5. Solid-state photoluminescence data of **Au_3**, **Au_6Ag**, and **Au_9Ag**.

Sample	λ_{ex} (nm)	λ_{em}(nm)	Φ_{PL} [a] [RT / 77 K] (%)	τ [b] (μs)
**Au_3**	351	670	67.5 / 78.8	15.32
**Au_6Ag**	354	496	43.9 / 95.2	9.31
**Au_9Ag**	370	496	12.0 / 39.1	9.11

[a] Absolute photoluminescence quantum yield. [b] Emission lifetime.

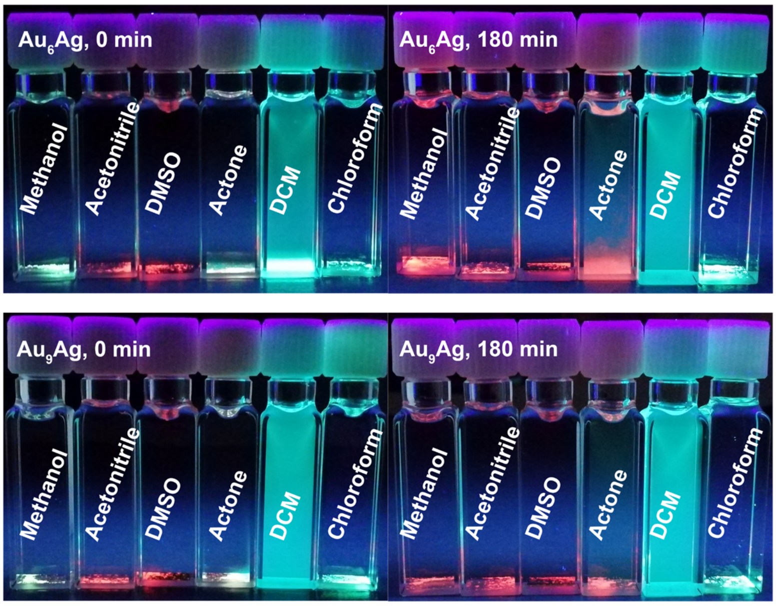

Figure S18. Digital photographs for the crystals of (top) **Au_6Ag** and (bottom) **Au_9Ag** immersing in different solvents (methanol, acetonitrile, DMSO, acetone, DCM, and chloroform, from left to right, respectively) under 365-nm hand-held UV lamp. It is clear to see that upon adding methanol, acetonitrile, DMSO, and acetone, the heterometallic Au/Ag clusters turns into white powder in a short time, assuming the lability of sandwich-like structures in high-polar solvents. Only DCM solution of both **Au_6Ag** and **Au_9Ag** could keep in a good shape for a long time period and the crystals remain well in chloroform.

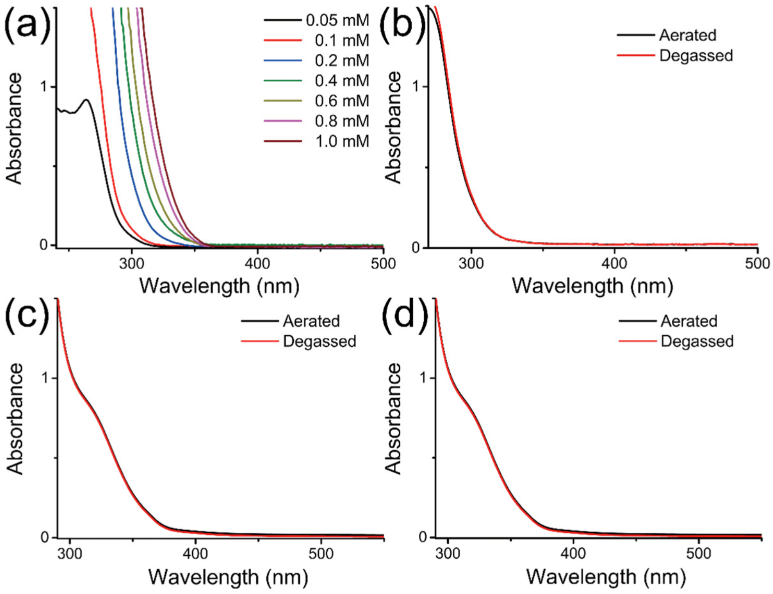

Figure S19. (a) Varied-concentration UV-vis absorption spectra of aerated **Au_3** CH_2Cl_2 solution. UV-vis absorption spectra of aerated and degassed 0.1 mM (b) **Au_3**, (c) **Au_6Ag**, and (d) **Au_9Ag** CH_2Cl_2 solution.

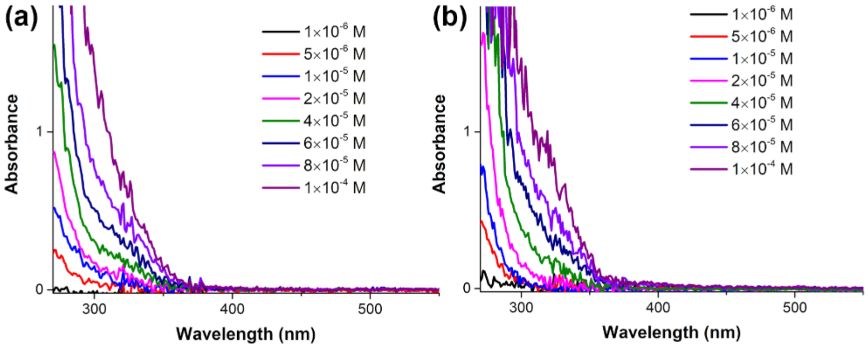

Figure S20. UV-vis absorption spectra for (a) **Au_6Ag** and (b) **Au_9Ag** at the concentrations ranging from 1×10^{-6} M to 1×10^{-4} M in CH_2Cl_2.

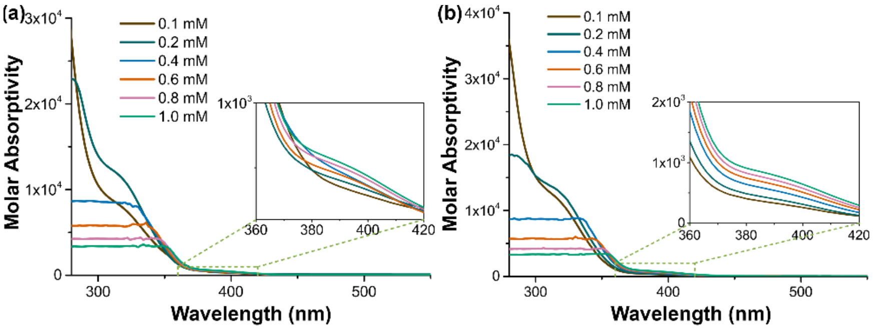

Figure S21. Molar absorptivity versus concentration of (a) **Au_6Ag** and (b) **Au_9Ag** CH_2Cl_2 solution at ambient temperature in the 10^{-4} M range. The inset zooms the wavelength region from 360 to 420 nm.

The aggregations of sandwich metal cluster in solutions give rise of deviation of Beer's Law, and oligomerization progress for **Au_xAg** (x = 6; 9) could be represented by eq. 1, with an equilibrium constant given by eq. 2. The differences between the highest absorption peak and low-energy absorption peak are as large as of 6,337 cm^{-1} for **Au_6Ag** and 6,225 cm^{-1} for **Au_9Ag**, which are also reasonable for monomer-dimer difference.

$$n[\mathbf{Au_xAg}] \rightleftharpoons [\mathbf{Au_xAg}]_n \qquad \text{eq. 1}$$

$$K_{1n} = c_n/c_1^n \qquad \text{eq. 2}$$

Based on original expression of Beer's Law and eq. 2, the following eq. 3 could be derived by assumption of the appearance of dimer in solutions. The fitting curves and parameters are enclosed in Figures 3b and S21c. Detailed derivation and expression could refer to M. A. Rawashdeh-Omary, M. A. Omary, H. H. Patterson, *J. Am. Chem. Soc.* **2000**, *122*, 10371-10380. c_0 is initial concentration; A is the absorbance for selected wavelength; ε_n is the molar extinction coefficient of the *n*-mer; b is the light path (1 cm here).

$$c_0 A^{-\frac{1}{n}} = \left(\frac{n}{\epsilon_n b}\right) A^{\frac{n-1}{n}} + (K_{1n}\epsilon_n b)^{-\frac{1}{n}} \qquad \text{eq. 3}$$

Table S6. Summary of equilibrium constants and free energies of **Au_6Ag** and **Au_9Ag** in CH_2Cl_2 solution at 298 K.

Species	Equilibrium constant K_{eq}	Free energy ΔG (298 K, kJ mol^{-1})
**Au_6Ag**	1.35×10^4 M^{-1}	-23.6
**Au_9Ag**	1.89×10^3 M^{-1}	-18.7

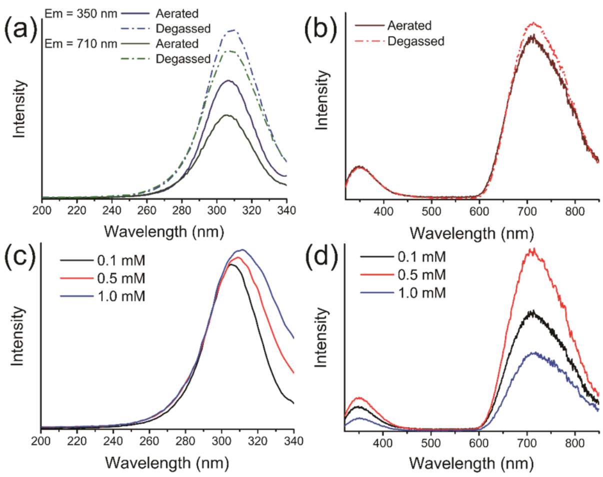

Figure S22. (a) Excitation spectra and (b) emission spectra of **Au_3** CH_2Cl_2 solution at the concentration of 0.1 mM in aerated and degassed situation. (c) Varied-concentration excitation spectra (Em = 710 nm) and (d) emission spectra (Ex = 310 nm) of aerated **Au_3** CH_2Cl_2 solution.

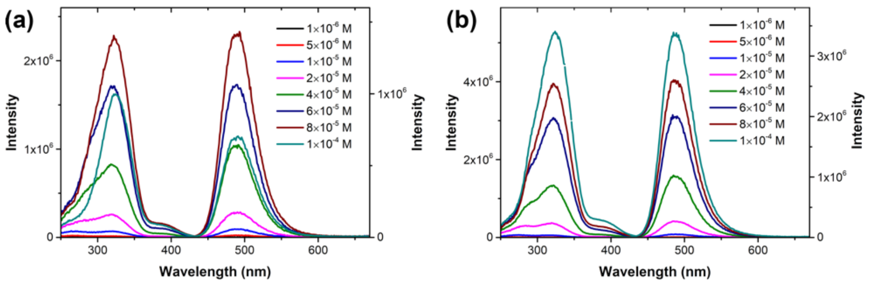

Figure S23. Varied-concentration excitation and emission spectra for (a) **Au_6Ag** and (b) **Au_9Ag** from the concentration range of 1×10^{-6} M to 1×10^{-4} M in CH_2Cl_2. The excitation wavelength is 300 nm and the emission wavelength is recorded at 490 nm.

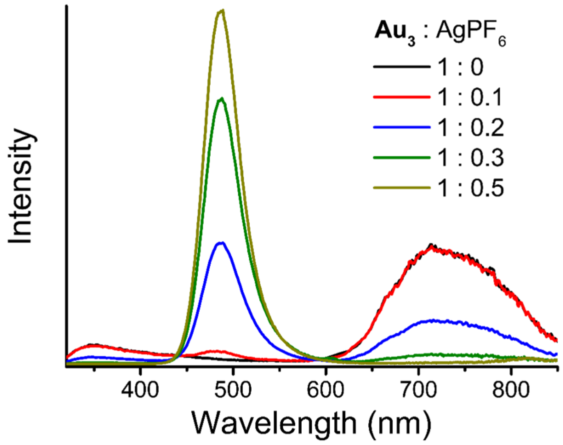

Figure S24. Emission Spectra of 0.1 mM different molar ratio of **Au_3**/$AgPF_6$ in CH_2Cl_2 solution (Ex = 310 nm).

Table S7. Absolute photoluminescence quantum yields (Φ_{PL}) of three nanoclusters in solid states.

Sample	Area of absorbance	Area of emission	Φ_{PL} / %	$\Phi_{PL, avg}$ / %	Sample	Area of absorbance	Area of emission	Φ_{PL} / %	$\Phi_{PL, avg}$ / %
**Au_6Ag**, 298 K	51610112	22682631	43.95	43.89 ± 0.16	**Au_6Ag**, 77 K	8465519	8078675	95.43	95.22 ± 0.19
	51517930	22674902	44.01			8395455	7981554	95.07	
	51452398	22486467	43.70			8402608	7995525	95.16	

**Au_9Ag**, 298 K	22127140	2665666	12.05	12.03 ± 0.09	**Au_9Ag**, 77 K	9447314	3664965	38.79	39.08 ± 0.26
	22106361	2674536	12.10			9318154	3661416	39.29	
	22111145	2637489	11.93			9335304	3656789	39.17	
**Au_3**, 298 K	22509314	15248334	67.74	67.48 ± 0.30	**Au_3**, 77 K	7750306	6042756	77.97	78.79 ± 0.96
	22660114	15306749	67.55			7577026	6050305	79.85	
	22661086	15217176	67.15			7709682	6056491	78.56	

Table S8. Absolute photoluminescence quantum yields (Φ_{PL}) of heterobimetallic nanoclusters in the solution.[a]

Concentration / mM	Area of absorbance	Area of emission	Φ_{PL} / %	$\Phi_{PL, avg}$ / %	Concentration / mM	Area of absorbance	Area of emission	Φ_{PL} / %	$\Phi_{PL, avg}$ / %
**Au_6Ag**									
0.01	74810814	4137316	5.53	5.52 ± 0.01	0.05	110889936	23026397	20.77	20.73 ± 0.03
	74917834	4132701	5.52			110855306	22973864	20.72	
	74809097	4121080	5.51			110884007	22963920	20.71	
0.1	140545799	37864526	26.94	26.96 ± 0.02	0.2	145816804	45979174	31.53	31.56 ± 0.04
	140561195	37919119	26.98			145811715	46012195	31.56	
	140547200	37870492	26.95			145830560	46089327	31.60	
0.4	146473898	62737211	42.83	42.80 ± 0.03	0.6	148048994	76656305	51.78	51.78 ± 0.05
	146488927	62662914	42.78			148052818	76608571	51.74	
	146486345	62672101	42.78			148064597	76740031	51.83	
0.8	146532232	73531006	50.18	50.19 ± 0.04	1.0	27485141	16621814	60.48	60.58 ± 0.17
	146546109	73618317	50.24			27432589	16672441	60.78	
	146527558	73494605	50.16			27488810	16627400	60.49	
0.1 (degassed)	127975892	32836773	25.66	25.36 ± 0.26					
	127834847	32248035	25.23						
	127832507	32207727	25.20						
**Au_9Ag**									
0.01	70562062	6211009	8.80	8.80 ± 0.02	0.05	126010235	30800673	24.44	24.43 ± 0.01
	70487697	6198576	8.79			126039089	30799129	24.44	
	70470152	6216310	8.82			126029092	30779483	24.42	
0.1	146903366	49927218	33.99	33.98 ± 0.02	0.2	147556632	53696703	36.39	36.37 ± 0.04
	146885952	49917962	33.98			147542547	53701998	36.40	
	146895562	49886777	33.96			147558690	53596887	36.32	
0.4	149221108	60992117	40.87	40.85 ± 0.07	0.6	150587343	54949514	36.49	36.43 ± 0.05
	149206952	61019425	40.90			150585131	54825400	36.41	
	149203433	60836211	40.77			150580602	54815382	36.40	
0.8	149332966	58985095	39.50	39.50 ± 0.04	1.0	147406353	50367480	34.17	34.15 ± 0.03
	149318215	58937893	39.47			147388025	50350616	34.16	
	149331323	59040148	39.54			147389249	50293912	34.12	
0.1 (degassed)	137833729	45051851	32.69	32.68 ± 0.01					
	137839714	45042703	32.68						
	137849121	45034393	32.67						

[a] All measurements were performed in undegassed dichloromethane solution under room temperature if not specifically informed.

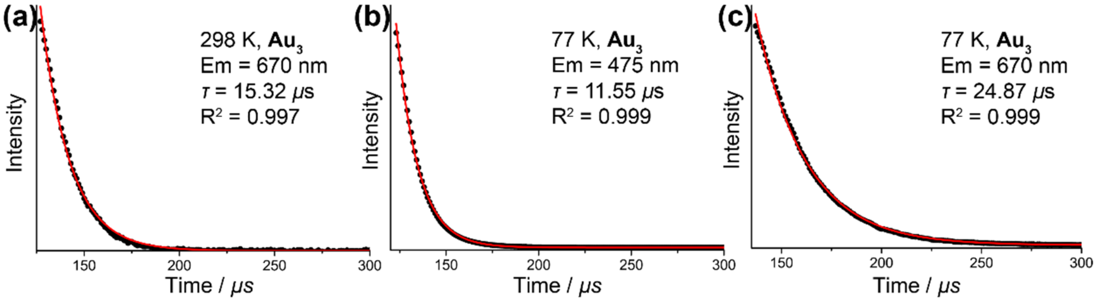

Figure S25. Emission decay profiles of **Au_3** in the solid state under (a) 298 K and (b-c) 77K.

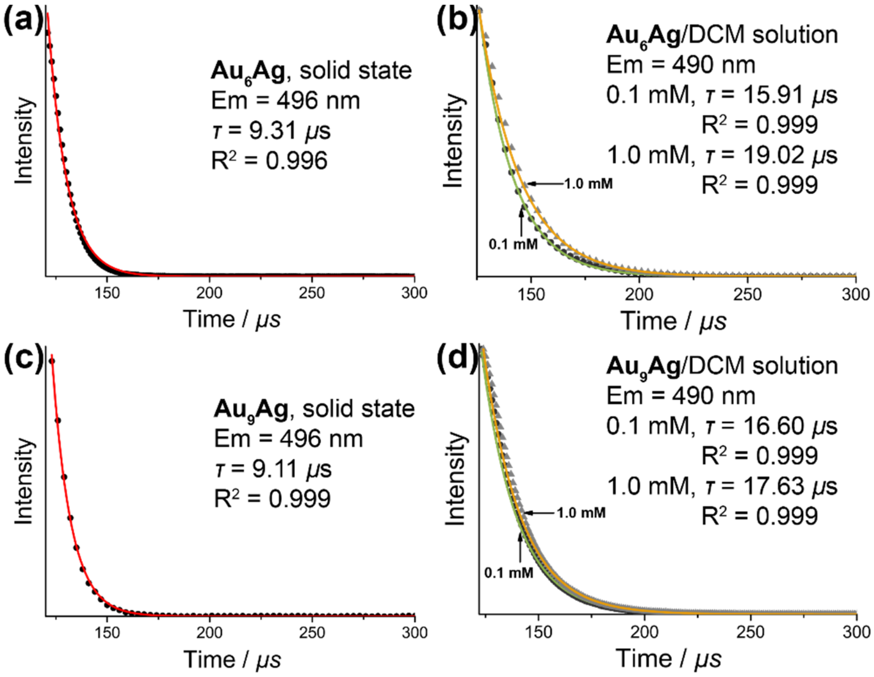

Figure S26. Emission decay profiles of (a-b) **Au_6Ag** and (c-d) **Au_9Ag** in the solid state and solution, respectively.

Computational Section

Computational Details

All initial models were taken from X-ray crystallography data. Dimer of **Au_3** was taken from crystal at 293 K with shortest inter-molecular Au-Au distance of 3.368 Å; both **Au_6Ag** and **Au_9Ag** units were taken from one set of disorder structure omitting counteranions, replacing ethoxycarbonyl with methoxycarbonyl groups since these parts have little contributions to electronic transitions and thus denoted as **Au_6Ag*** and **Au_9Ag***, respectively. Geometry optimizations were performed by Amsterdam Modeling Suite (AMS 2018) program package[1] at the level of Grimme's D3-dispersion-corrected[2] PBE functional[3,4] with Becke-Johnson (BJ) damping[5] and slater-type triple-zeta polarity (TZP) basis set,[6] using small frozen core approximation. No imaginary frequencies were observed in all optimized structures to confirm the minima on the energy surface. Energy decomposition analysis-natural orbitals for chemical valence (EDA-NOCV)[7-9] calculations were conducted at the level of PBE-D3(BJ) functional and TZP basis sets with none frozen core, in which the interaction energies (ΔE_{int}) between two fragments were decomposed to Pauli repulsions (ΔE_{Pauli}), electrostatic interactions (ΔE_{Elstat}), orbital interactions (ΔE_{Orb}), and dispersion correction energies (ΔE_{Disp}). Relativistic effects were considered by apply zeroth-order regular approximation (ZORA) to the full relativistic effect treated in Dirac equation.[10]

Time-dependent density functional theory (TDDFT) calculations were performed by Gaussian 16 A.03 software package[11] using PBE0 functional[12,13] with the effective core potential (ECP) of Lanl2dz basis set[14,15] for metal atoms and 6-31G(d,p) basis set[16] for non-metal atoms. The first 20 $S_0 \rightarrow S_n$ and first 10 $S_0 \rightarrow T_n$ vertical transitions were calculated for each model. All molecular orbital maps and electron density difference (EDD) maps of singlet-singlet spin-allowed ($S_0 \rightarrow S_n$) and singlet-triplet spin-forbidden ($S_0 \rightarrow T_n$) transitions were generated by Multiwfn 3.6 software[17] using the formatted checkpoint file (.fchk files) and the Gaussian output file (.out files). The isovalue of contour is 0.02 a.u. for molecular orbitals (MOs) and 5×10^{-4} a.u. for EDA-NOCV deformation density and EDD maps. Abbreviations in this section: *f*, oscillator strength; HOMO or H, highest occupied molecular orbital; LUMO or L, lowest unoccupied molecular orbital; MLCT, metal-to-ligand charge transfer; ILCT, inter-ligand charge transfer; MC, metal-centred.

Computational Results

Scheme S1. Energy level diagram for all four models.

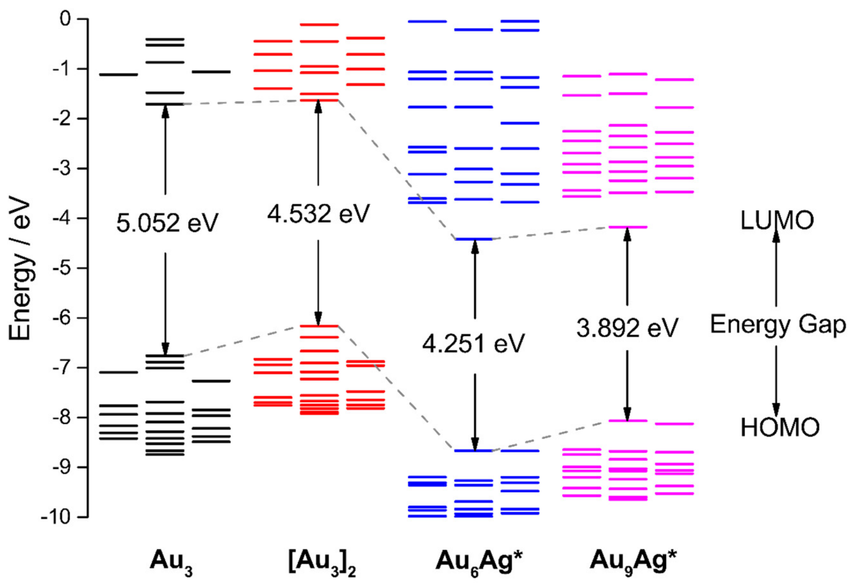

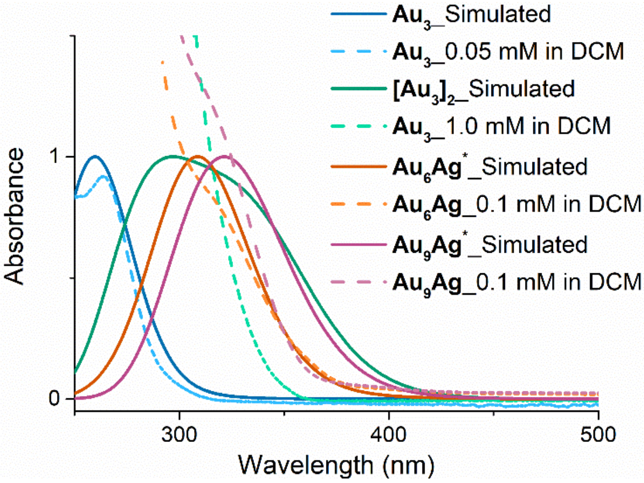

Figure S27. Comparison of experimental and simulated UV-vis absorption spectra for **Au_3**, **$[Au_3]_2$**, **Au_6Ag***, and **Au_9Ag***.

Table S9. Energy decomposition analysis (EDA) results for heterobimetallic nanocluster. (unit: kcal/mol)

	**Au_6Ag***		**Au_9Ag***		
Fragment 1	[**Au_3**]$_2$*	**Au_3***Ag^+	[**Au_3**]$_3$*	[**Au_3**]$_2$*	[**Au_3**]*Ag^+[**Au_3**]*
Fragment 2	Ag^+	**Au_3***	Ag^+	**Au_3***Ag^+	**Au_3***
ΔE_{Pauli}	128.0	131.2	125.3	135.4	101.0
ΔE_{Elstat}	-134.4 (52.2%)	-110.2 (52.1%)	-132.4 (51.1%)	-114.1 (50.6%)	-75.7 (47.5%)
ΔE_{Orb}	-110.6 (43.0%)	-56.7 (26.8%)	-113.2 (43.7%)	-60.3 (26.8%)	-33.1 (20.7%)
ΔE_{Disp}	-12.3 (4.8%)	-44.7 (21.1%)	-13.5 (5.2%)	-51.0 (22.6%)	-50.7 (31.8%)
ΔE_{Int}	-129.3	-80.4	-133.8	-89.1	-58.5

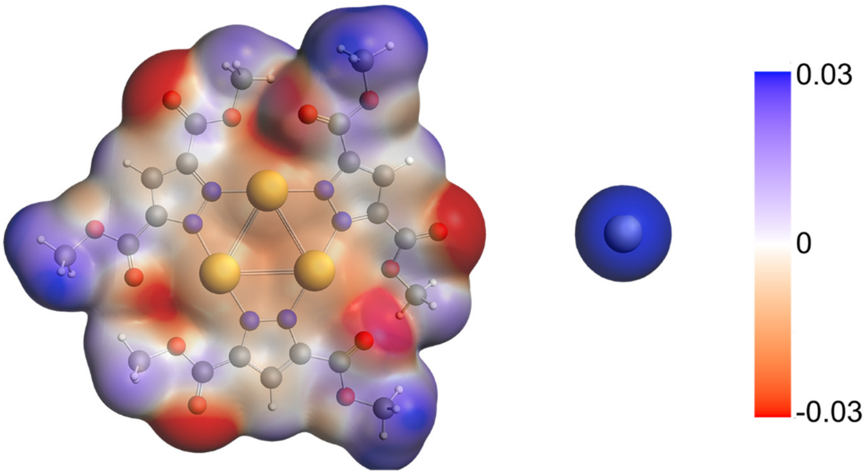

Figure S28. Electrostatic potential (ESP) maps for (left) **Au_3** and (right) Ag^+ species.

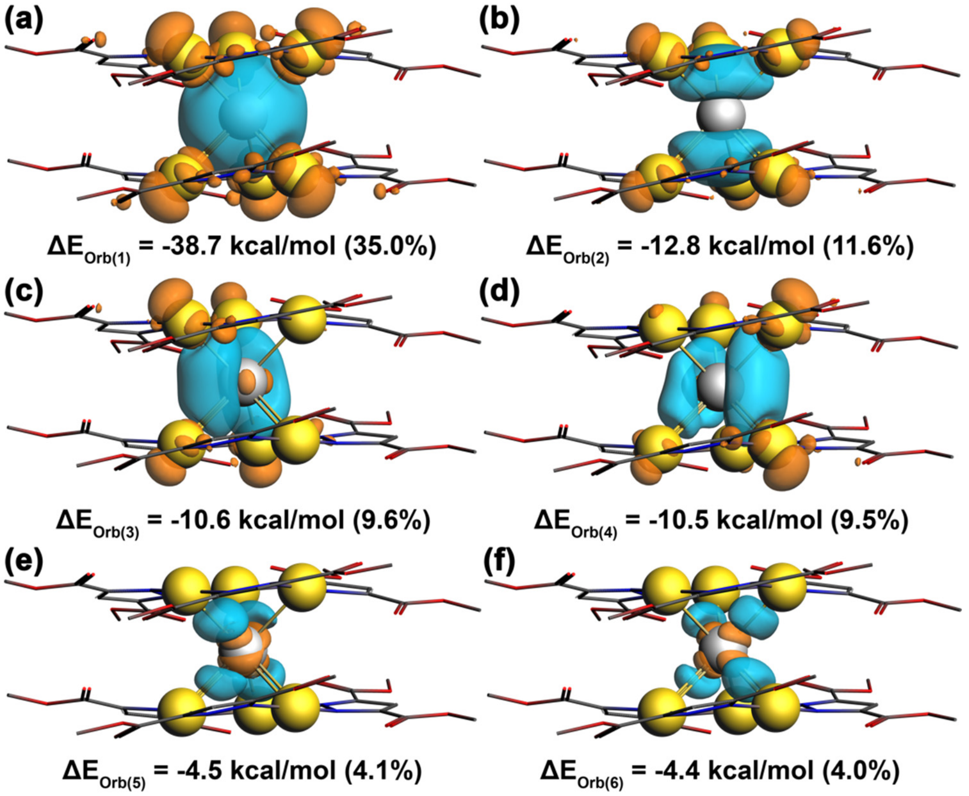

Figure S29. Shape of the EDA-NOCV deformation densities (a-f) $\Delta\rho_{(1)\text{-}(6)}$, which are also associated with the orbital interaction $\Delta E_{orb(1)\text{-}(6)}$ in **Au₆Ag*** and the contribution thereof (in parentheses). The model of **Au₆Ag*** (charge: +1) here are separated as Ag^{+} and **[Au₃]₂***. The colour code of the electron flow is orange to cyan.

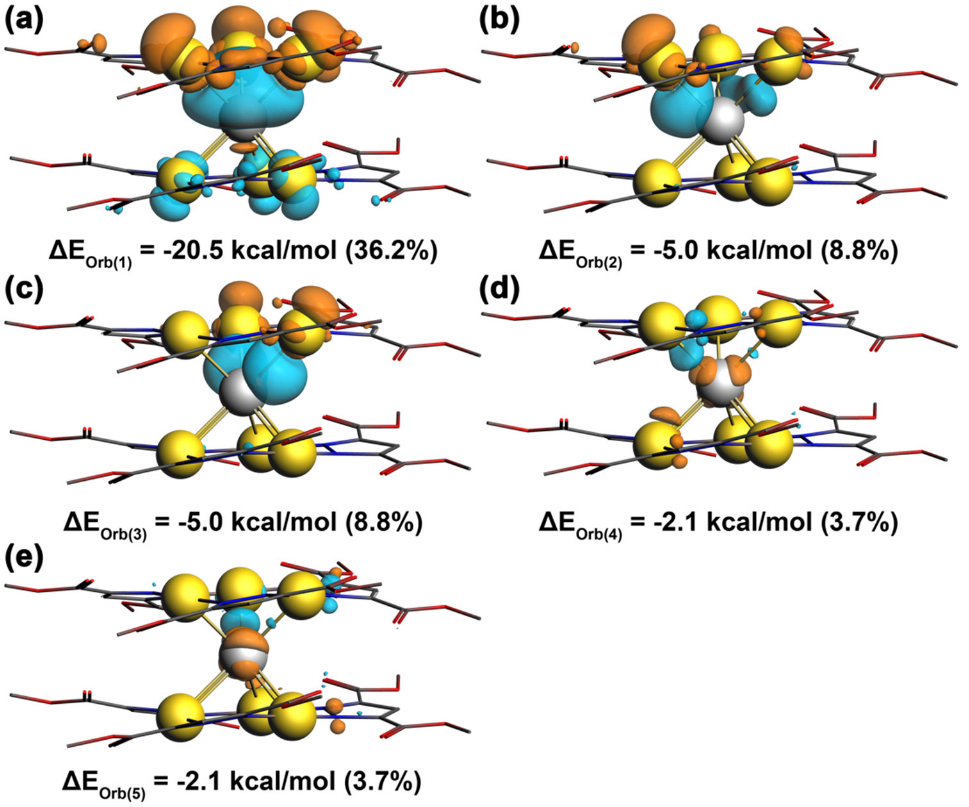

Figure S30. Shape of the EDA-NOCV deformation densities (a-e) $\Delta\rho_{(1)\text{-}(5)}$, which are also associated with the orbital interaction $\Delta E_{orb(1)\text{-}(5)}$ in **Au₆Ag*** and the contribution thereof (in parentheses). The model of **Au₆Ag*** (charge: +1) here are separated as **Au₃***Ag^{+} and **Au₃***. The colour code of the electron flow is orange to cyan.

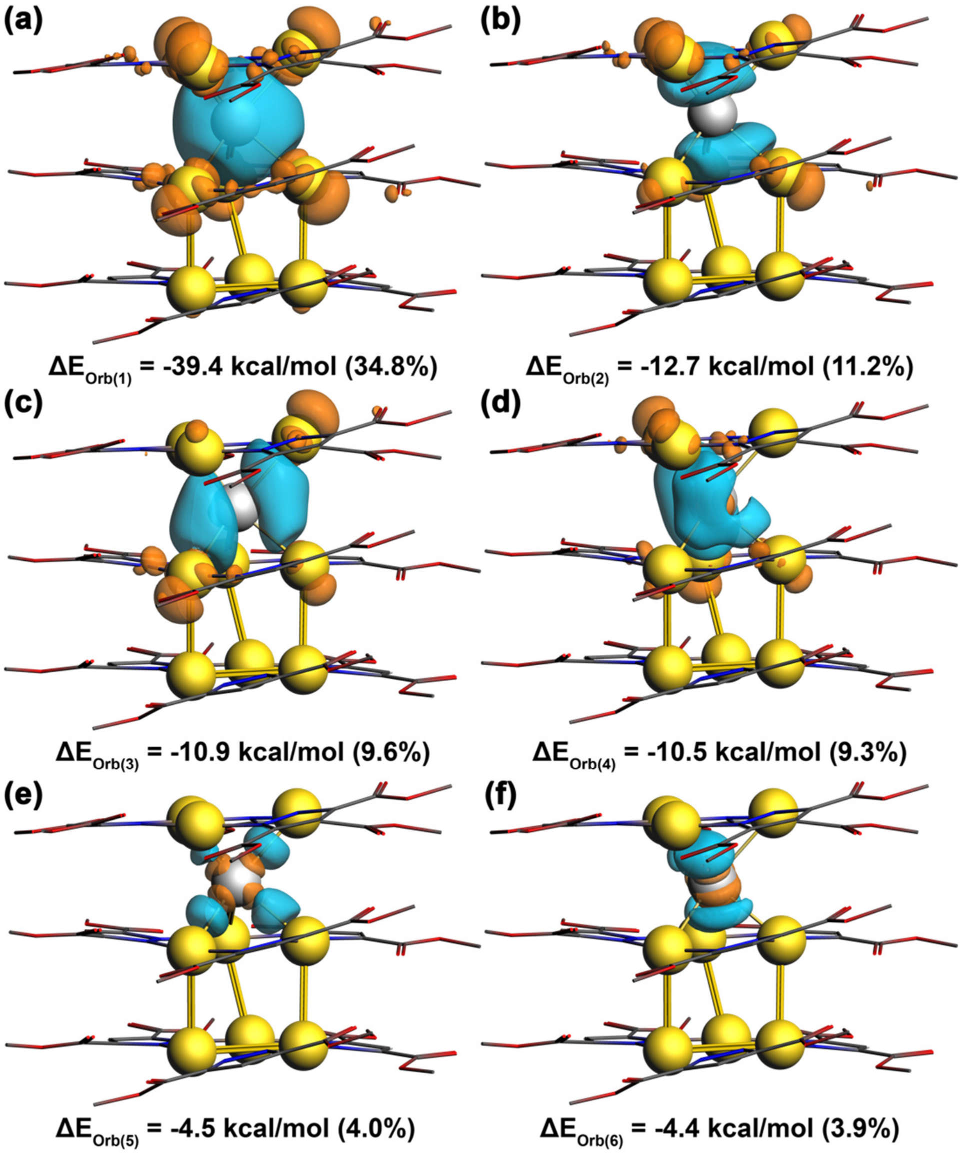

Figure S31. Shape of the EDA-NOCV deformation densities (a-f) $\Delta\rho_{(1)\text{-}(6)}$, which are also associated with the orbital interaction $\Delta E_{orb(1)\text{-}(6)}$ in **Au_9Ag*** and the contribution thereof (in parentheses). The model of **Au_9Ag*** (charge: +1) here are separated as Ag^+ and **$[Au_3]_3$***. The colour code of the electron flow is orange to cyan.

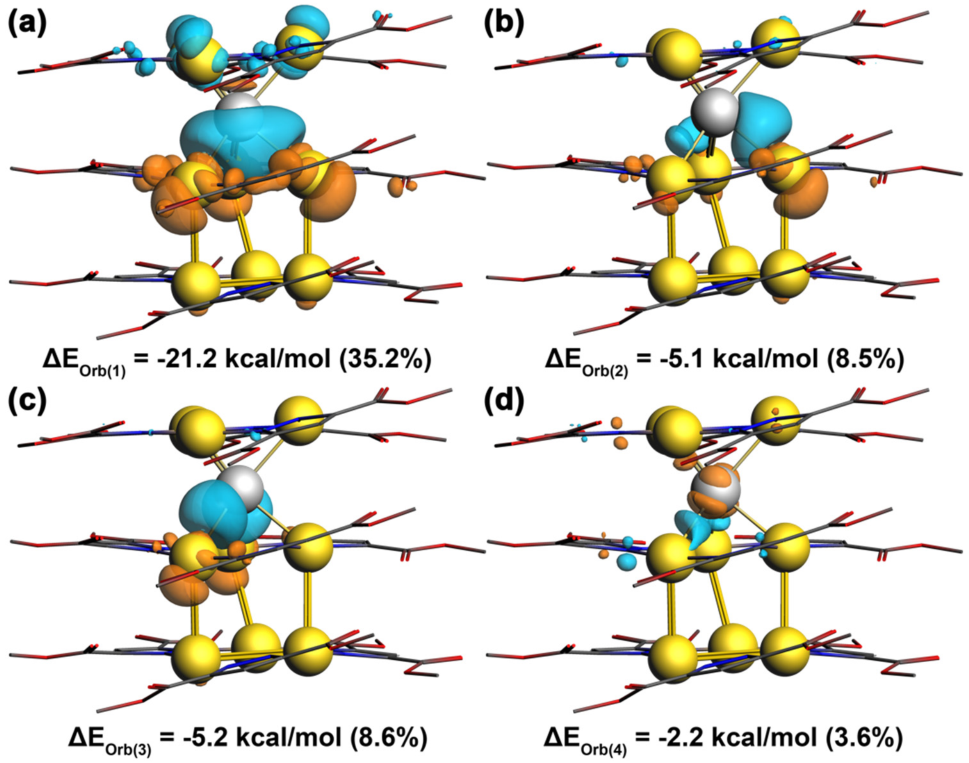

Figure S32. Shape of the EDA-NOCV deformation densities (a-d) $\Delta\rho_{(1)\text{-}(4)}$, which are also associated with the orbital interaction $\Delta E_{orb(1)\text{-}(4)}$ in **Au₉Ag*** and the contribution thereof (in parentheses). The model of **Au₉Ag*** (charge: +1) here are separated as **Au₃***Ag^+ and **[Au₃]₂***. The colour code of the electron flow is orange to cyan.

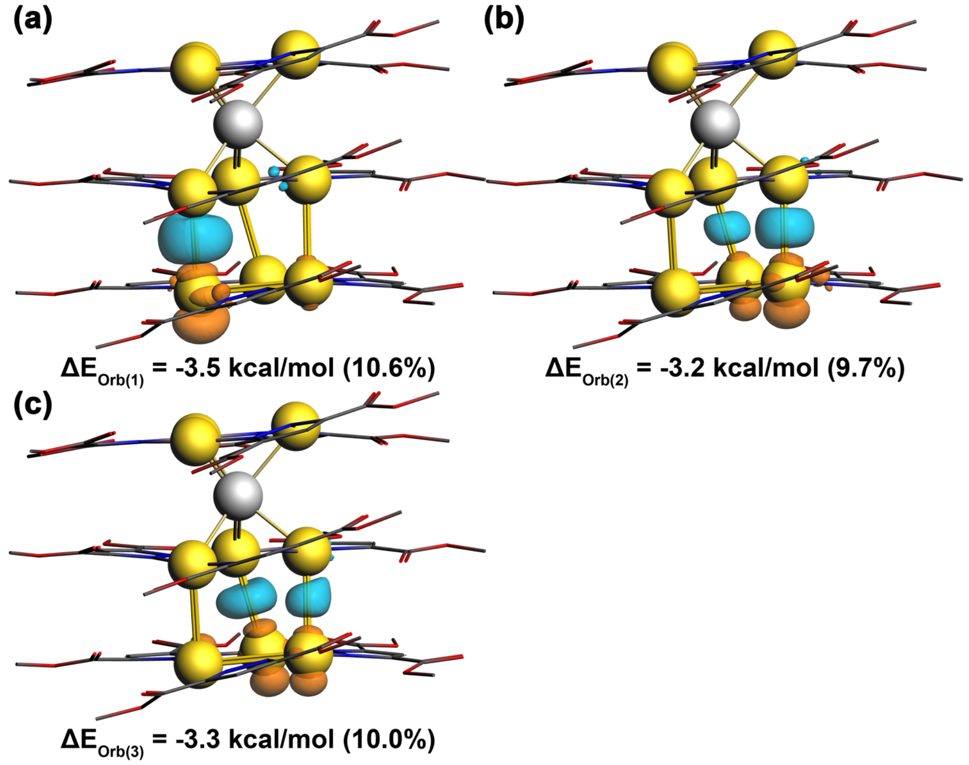

Figure S33. Shape of the EDA-NOCV deformation densities (a-c) $\Delta\rho_{(1)\text{-}(3)}$, which are also associated with the orbital interaction $\Delta E_{orb(1)\text{-}(3)}$ in **Au₉Ag*** and the contribution thereof (in parentheses). The model of **Au₉Ag*** (charge: +1) here are separated as [**Au₃**]*Ag^+[**Au₃**]*and **Au₃**. The colour code of the electron flow is orange to cyan.

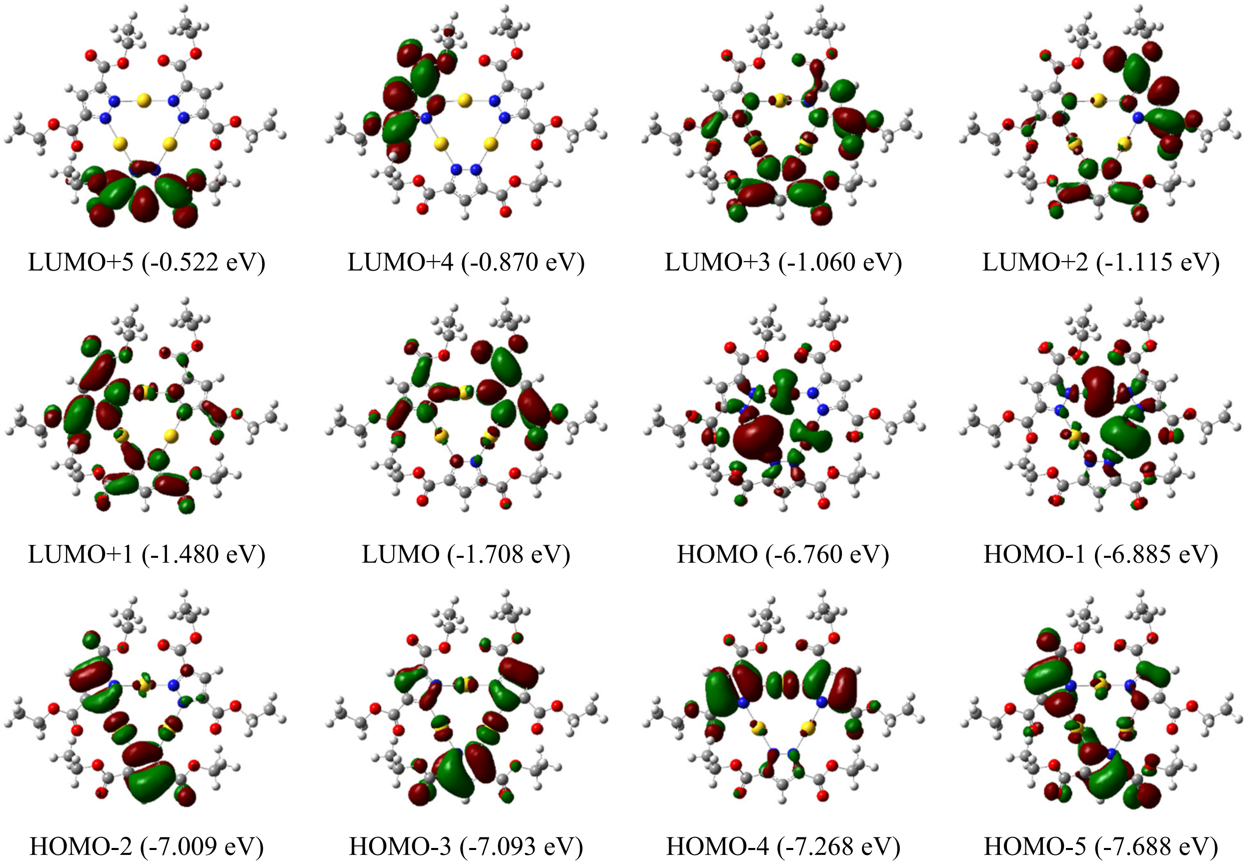

Figure S34. Selected molecular orbitals and energy level (eV) for monomer of **Au_3**.

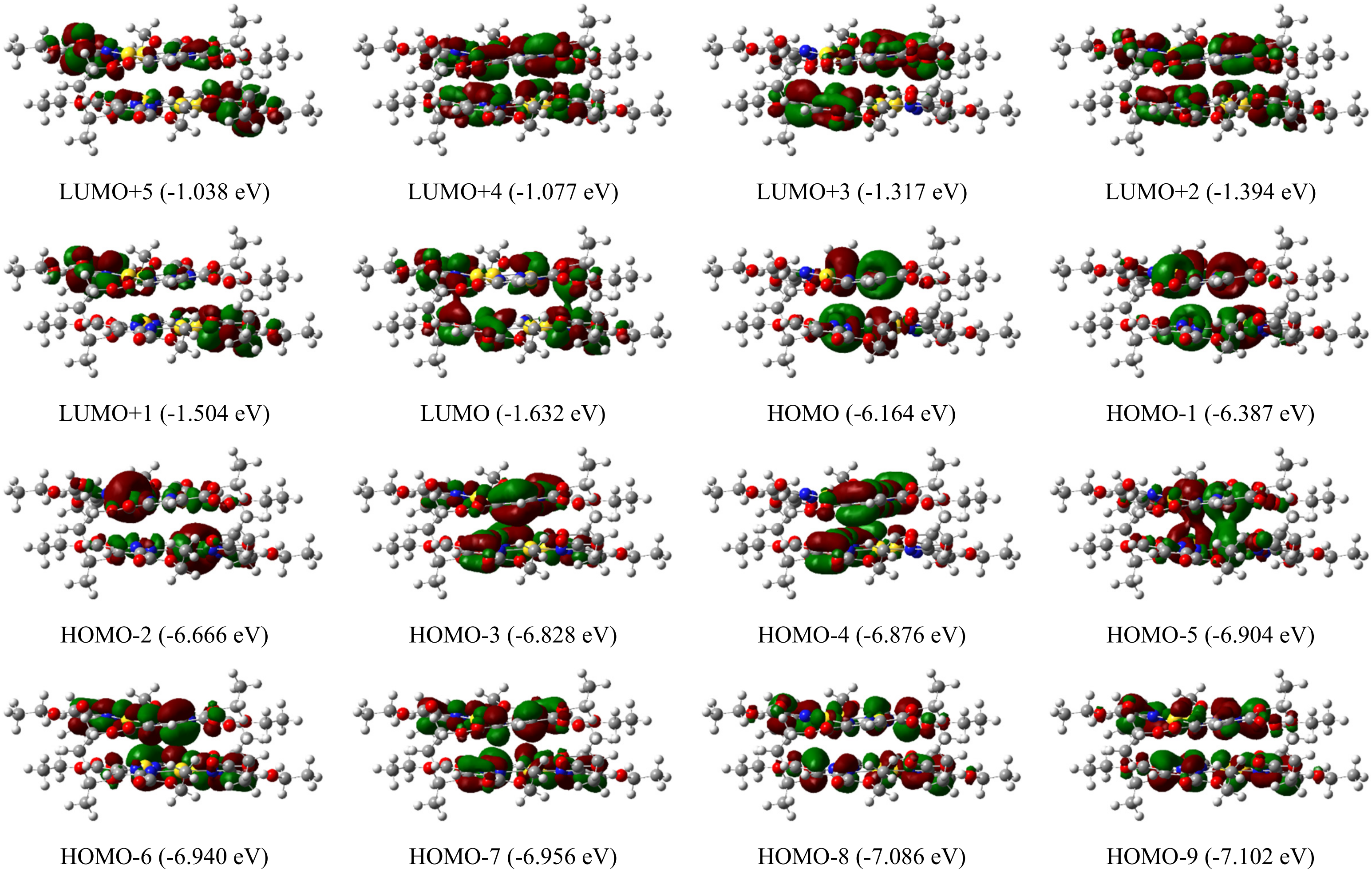

Figure S35. Selected molecular orbitals and energy level (eV) for dimer of **Au_3**.

Table S10. TD-DFT results of selected singlet excited states for monomer of **Au3**.

No.	E /ev (λ /nm)	EDD	*f*	Major transitions and contributions	Type
1	4.061 (305.5)	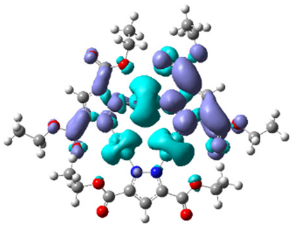	0.004	H-1→L (75%), H→L (13%), H→L+1 (6%)	^{1}MLCT
5	4.574 (271.2)	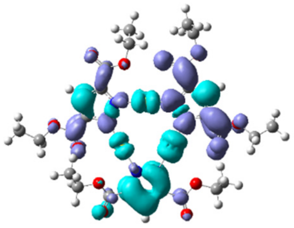	0.013	H-3→L (57%), H-2→L+1 (19%), H-4→L (12%)	^{1}MLCT/ ^{1}ILCT
7	4.651 (266.8)	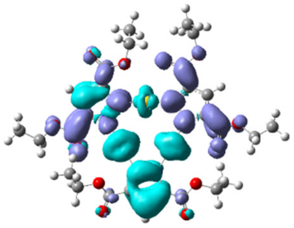	0.142	H-2→L (55%), H-2→L+1 (10%), H→L+3 (6%)	^{1}MLCT/ ^{1}ILCT
8	4.753 (261.0)	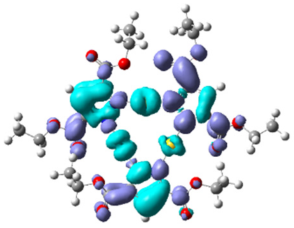	0.303	H-2→L+1 (50%), H-4→L (28%), H-2→L (7%)	^{1}MLCT/ ^{1}ILCT
9	4.785 (259.3)	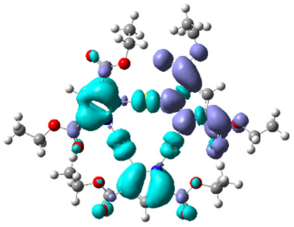	0.170	H-4→L (47%), H-3→L (23%), H-2→L (15%), H-2→L+1 (6%)	^{1}MLCT/ ^{1}ILCT

Table S11. TD-DFT results of selected singlet excited states for dimer of **Au3**.

No.	E /ev (λ /nm)	EDD		*f*	Major transitions and contributions	Type
		Top View	Side View			
1	3.662 (338.8)	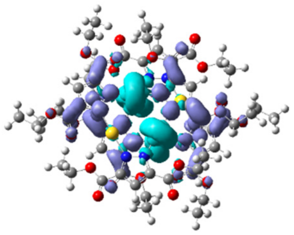	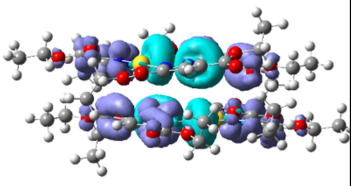	0.034	H→L (91%)	^{1}MLCT

2	3.777 (328.5)	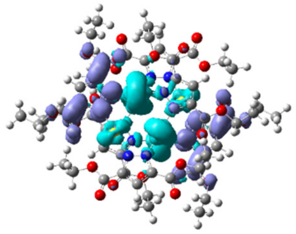	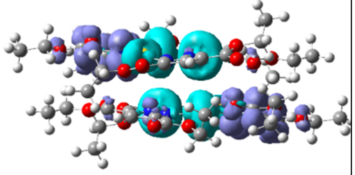	0.000	H→L+1 (54%), H-1→L (33%)	^{1}MLCT
3	3.868 (320.7)	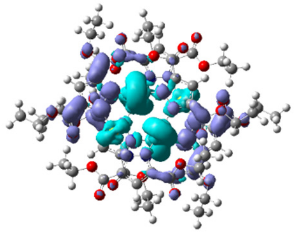	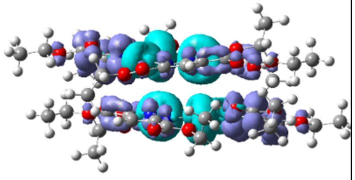	0.002	H→L+2 (59%), H-1→L+1 (22%), H-2→L (7%)	^{1}MLCT
6	4.011 (309.3)	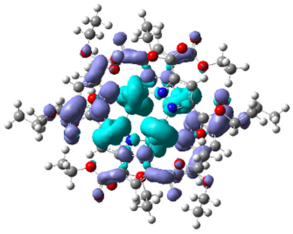	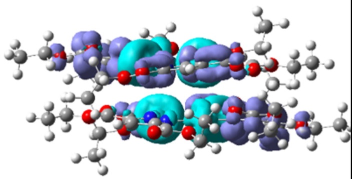	0.015	H-1→L+1 (38%), H→L+2 (29%), H-2→L (14%), H-2→L+2 (6%)	^{1}MLCT

Table S12. TD-DFT results of selected triplet excited states for monomer of **Au_3**.

No.	E /ev (λ /nm)	EDD	Major transitions and contributions	Type
1	3.519 (352.6)	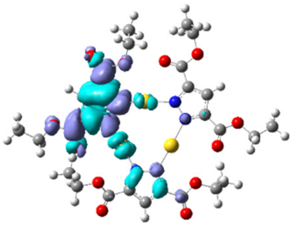	H-2→L+1 (19%), H-4→L+1 (18%), H-4→L (8%), H-5→L+1 (7%)	^{3}ILCT/ ^{3}MLCT
2	3.534 (351.1)	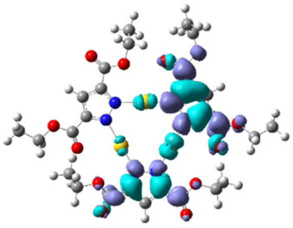	H-4→L (19%), H-9→L (14%), H-3→L (11%), H-3→L+1 (8%), H-3→L+3 (6%)	^{3}ILCT/ ^{3}MLCT
3	3.558 (348.7)	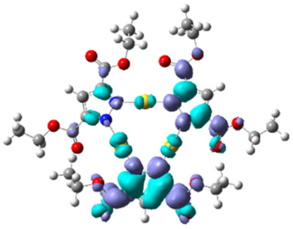	H-3→L+3 (14%), H-3→L+2 (8%), H-3→L+1 (6%), H-2→L+3 (6%)	^{3}ILCT/ ^{3}MLCT

Table S13. TD-DFT results of selected triplet excited states for dimer of **Au_3**.

No.	E /ev (λ /nm)	EDD		Major transitions and contributions	Type
1	3.445 (360.2)	Top View 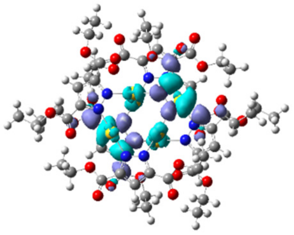	Side View 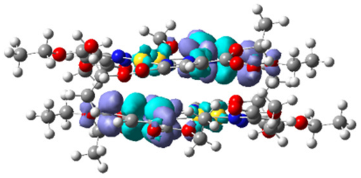	H→L (22%), H-9→L+3 (9%), H-8→L (9%)	^{3}ILCT/ ^{3}MLCT
2	3.462 (358.4)	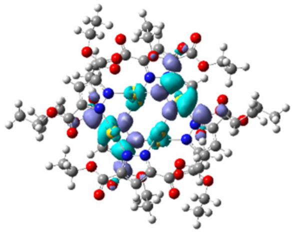	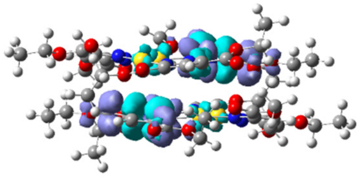	H-8→L+3 (13%), H-9→L (11%), H-3→L (6%)	^{3}ILCT/ ^{3}MLCT
3	3.504 (354.1)	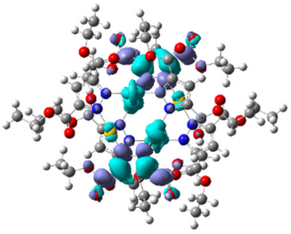	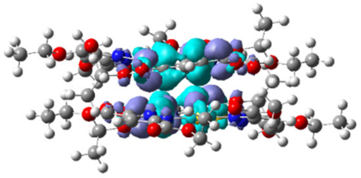	H-6→L+2 (9%), H→L (9%), H-7→L+4 (8%), H-4→L+2 (7%), H-7→L+3 (5%)	^{3}ILCT/ ^{3}MLCT

Au6Ag*

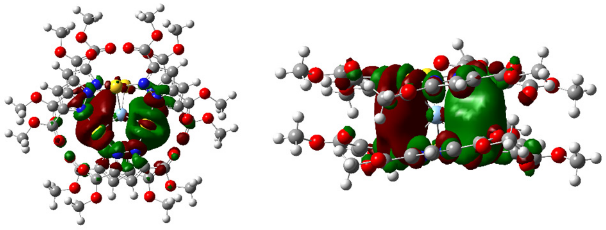

LUMO+16 (-1.768 eV)

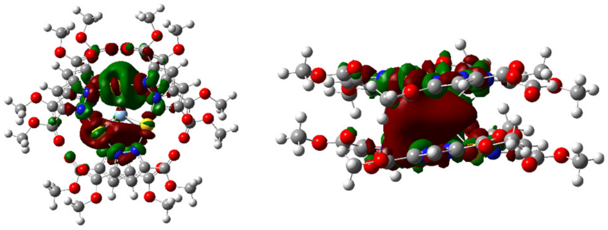

LUMO+15 (-1.769 eV)

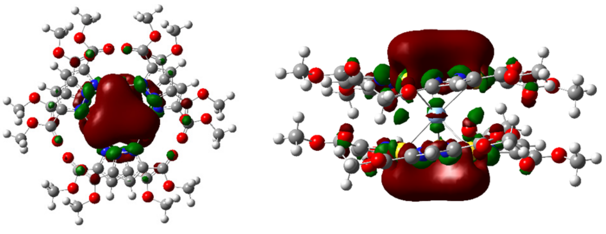

LUMO+14 (-2.089 eV)

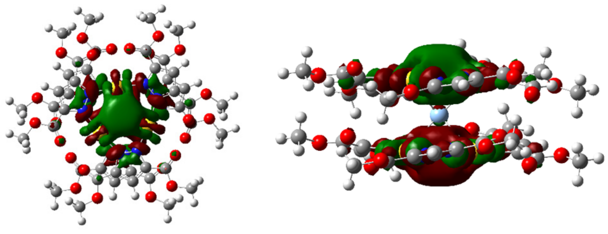

LUMO+10 (-2.671 eV)

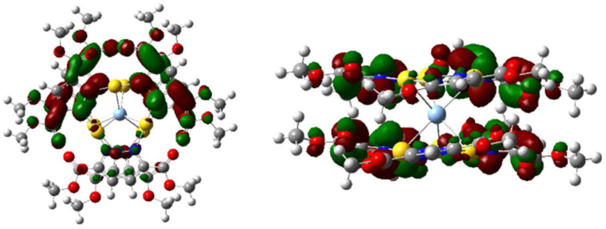

LUMO+3 (-3.619 eV)

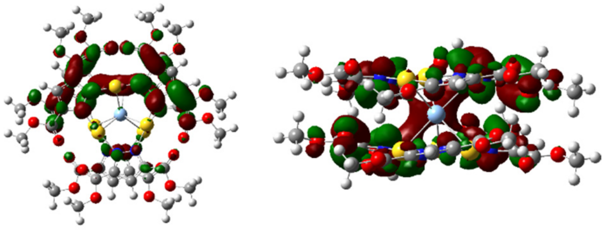

LUMO+2 (-3.677 eV)

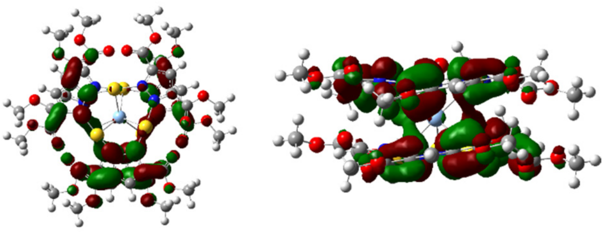

LUMO+1 (-3.687 eV)

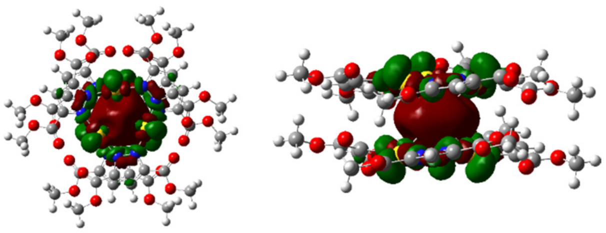

LUMO (-4.416 eV)

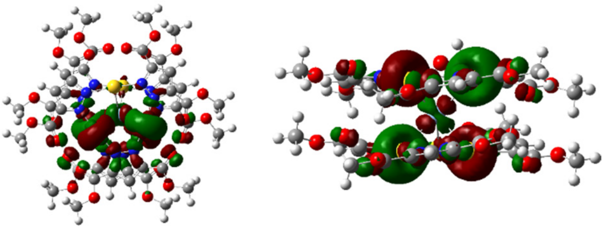

HOMO (-8.667 eV)

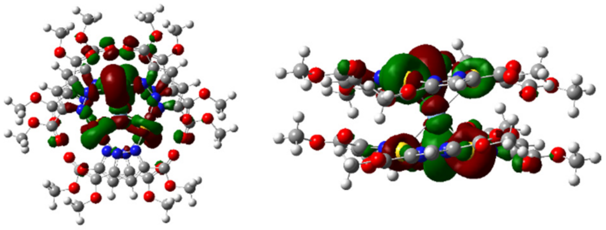

HOMO-1 (-8.673 eV)

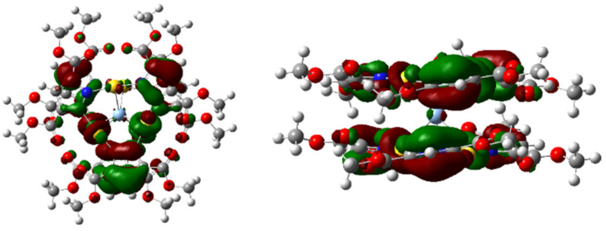

HOMO-2 (-9.194 eV)

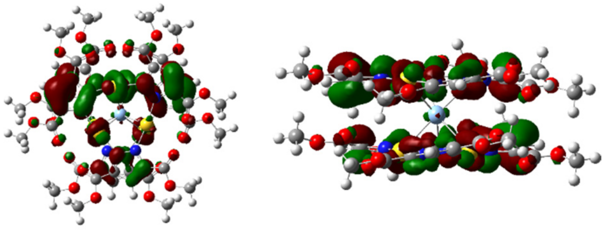

HOMO-3 (-9.198 eV)

Figure S36. Selected molecular orbitals and energy level (eV) for **Au_6Ag***.

Au9Ag*

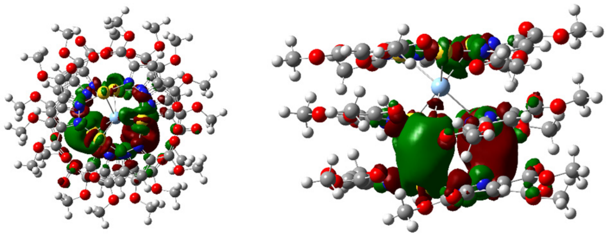

LUMO+26 (-1.106 eV)

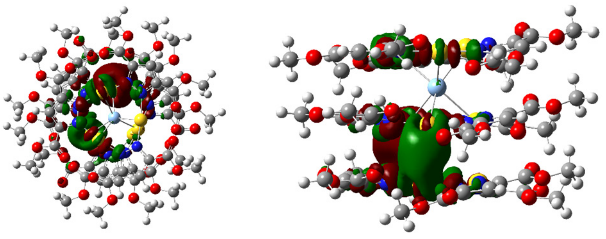

LUMO+25 (-1.149 eV)

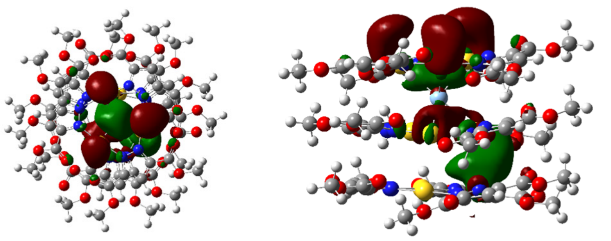

LUMO+24 (-1.217 eV)

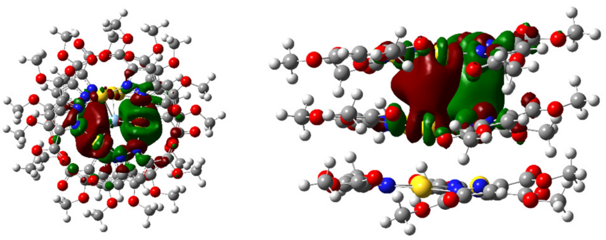

LUMO+23 (-1.500 eV)

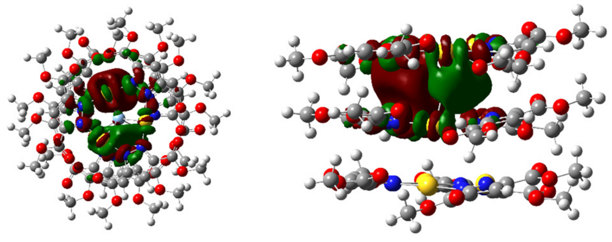

LUMO+22 (-1.532 eV)

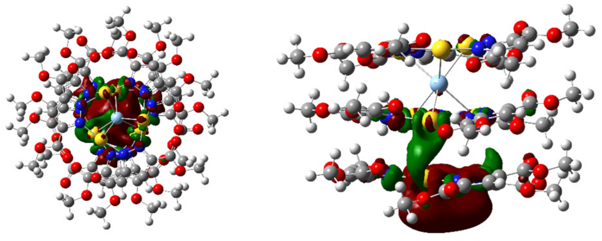

LUMO+21 (-1.775 eV)

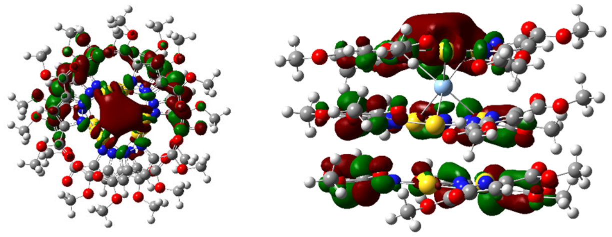

LUMO+17 (-2.350 eV)

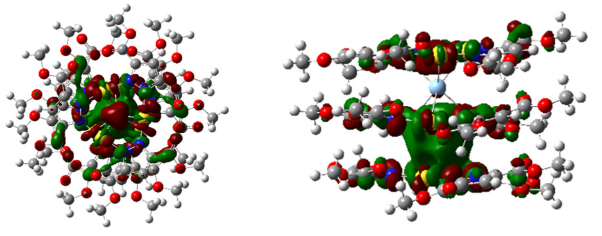

LUMO+13 (-2.690 eV)

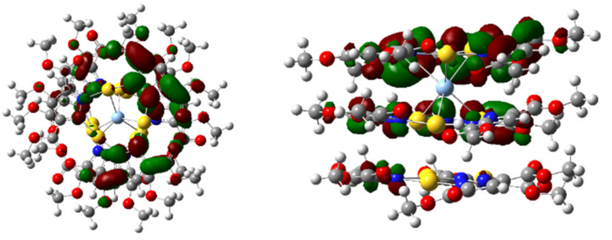

LUMO+3 (-3.471 eV)

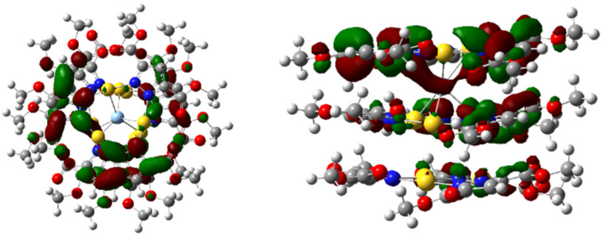

LUMO+2 (-3.486 eV)

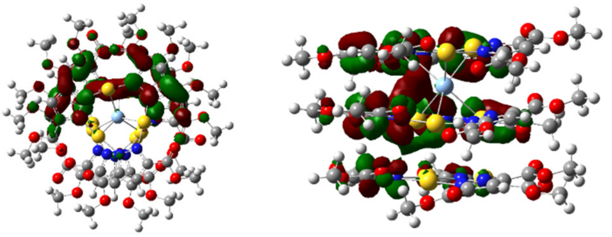

LUMO+1 (-3.562 eV)

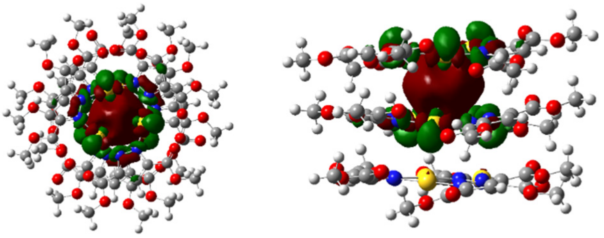

LUMO (-4.175 eV)

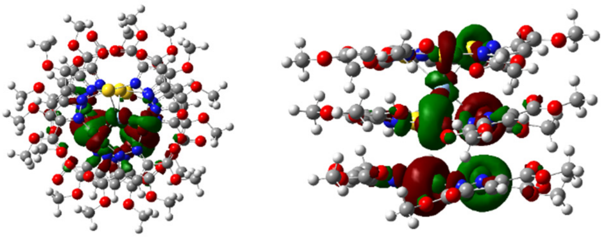

HOMO (-8.067 eV)

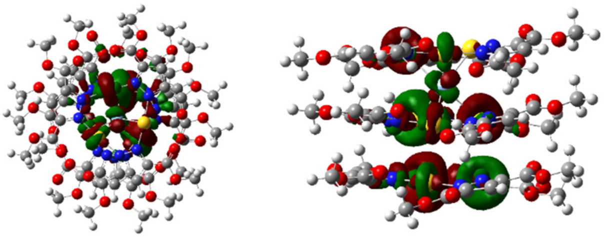

HOMO-1 (-8.126 eV)

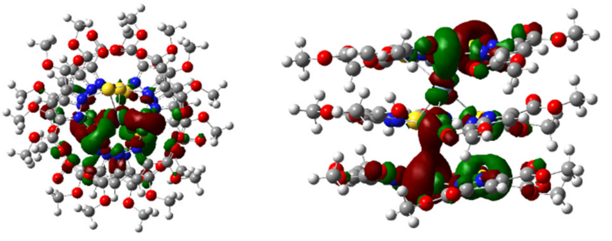

HOMO-2 (-8.645 eV)

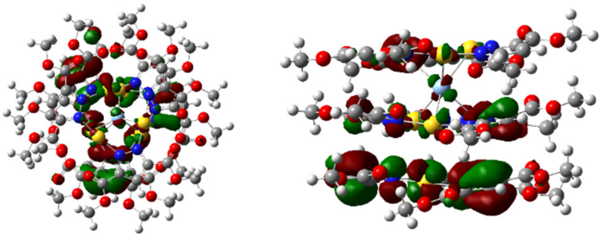

HOMO-3 (-8.676 eV)

Figure S37. Selected molecular orbitals and energy level (eV) for **Au_9Ag***.

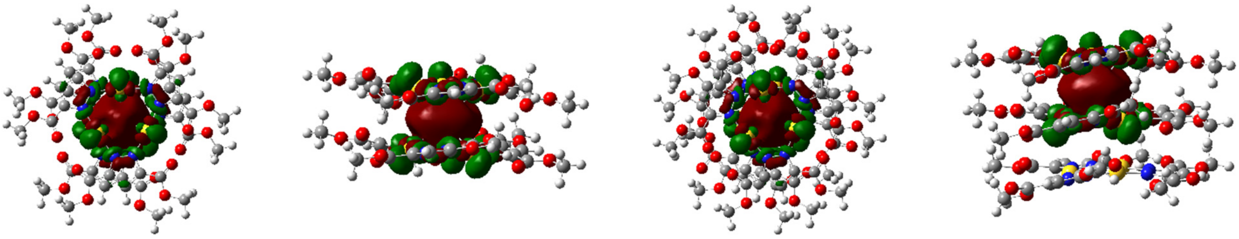

Au$_6$Ag* **Au$_9$Ag***

Figure S38. Highest singly occupied molecular orbitals (HSOMO) for **Au$_6$Ag*** and **Au$_9$Ag*** at T_1 state.

Table S14. TD-DFT results of selected singlet excited states for **Au$_6$Ag***.

No.	E /ev (λ /nm)	EDD		*f*	Major transitions and contributions	Type
		Top View	Side View			
1	3.345 (370.9)	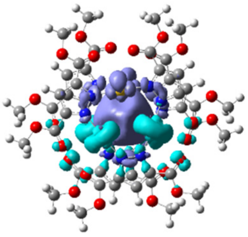	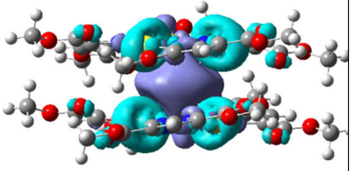	0.001	H→L (97%)	^{1}MC
2	3.350 (370.3)	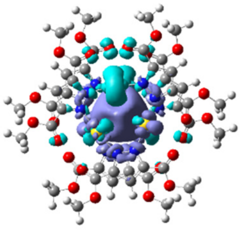	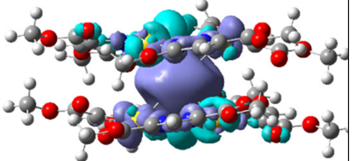	0.001	H-1→L (97%)	^{1}MC
6	3.955 (313.7)	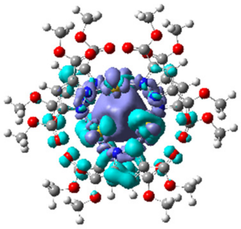	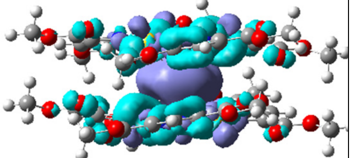	0.031	H-6→L (70%), H-5→L (20%)	^{1}LMCT/ ^{1}MC
7	3.956 (313.6)	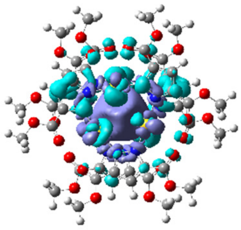	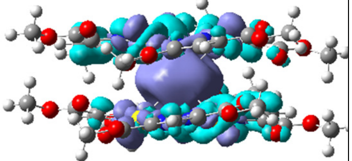	0.031	H-6→L (20%), H-5→L (69%)	^{1}LMCT/ ^{1}MC

Table S15. TD-DFT results of selected singlet excited states for **Au_9Ag***.

No.	E /ev (λ /nm)	EDD Top View	EDD Side View	*f*	Major transitions and contributions	Type
1	3.080 (402.9)	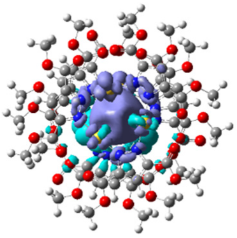	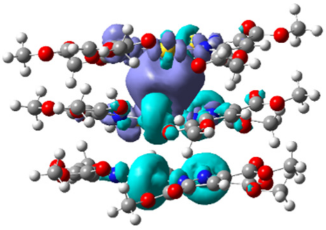	0.000	H→L (96%)	^{1}LMCT/ ^{1}MC
2	3.131 (396.3)	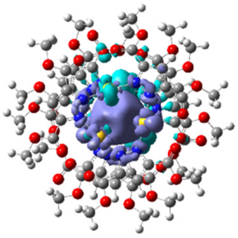	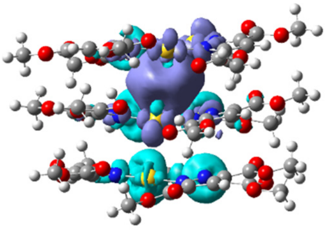	0.001	H-1→L (95%)	^{1}LMCT/ ^{1}MC
5	3.707 (334.7)	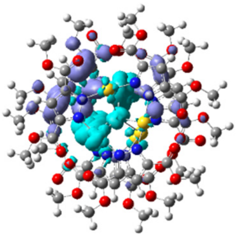	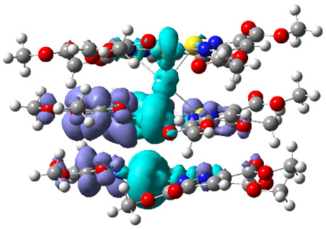	0.060	H-1→L+1 (32%), H→L+1 (31%), H→L+4 (9%), H-1→L+3 (6%), H→L+3 (4%)	^{1}MLCT
6	3.730 (332.7)	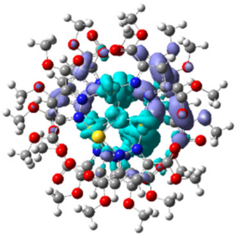	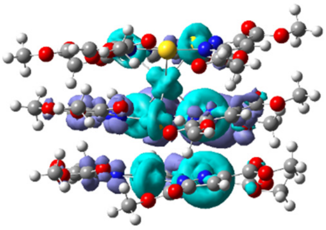	0.028	H→L+1 (32%), H-1→L+1 (27%), H-1→L+2 (12%), H→L+3 (7%), H-1→L+3 (6%)	^{1}MLCT

Table S16. TD-DFT results of selected triplet excited states for **Au_6Ag***.

No.	E /ev (λ /nm)	EDD Top View	EDD Side View	Major transitions and contributions	Type
1	2.877 (431.3)	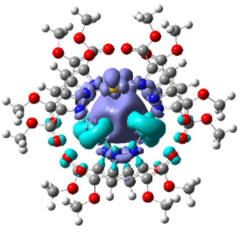	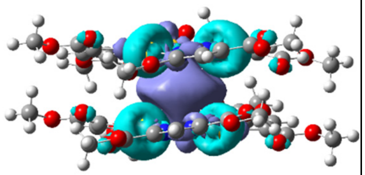	H→L (95%)	^{3}MC
2	2.884 (430.2)	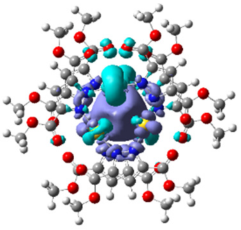	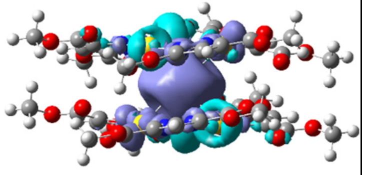	H-1→L (95%)	^{3}MC

Table S17. TD-DFT results of selected triplet excited states for **Au_9Ag***.

No.	E /ev (λ /nm)	EDD		Major transitions and contributions	Type
		Top View	Side View		
1	2.718 (456.4)	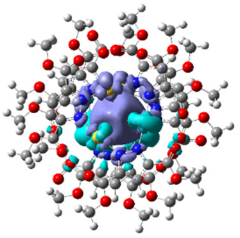	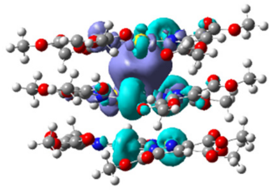	H→L (81%)	^{3}MC
2	2.767 (448.4)	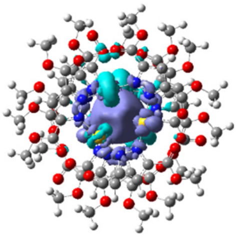	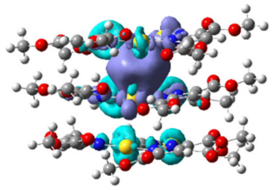	H-1→L (82%)	^{3}MC

References

(1) *ADF 2018*. SCM, Theoretical Chemistry, Vrije Universiteit, Amsterdam, The Netherlands, https://www.scm.com, **2018**.

(2) Grimme, S.; Antony, J.; Ehrlich, S.; Krieg, H. A Consistent and Accurate Ab Initio Parametrization of Density Functional Dispersion Correction (DFT-D) for the 94 Elements H-Pu. *J. Chem. Phys.* **2010**, *132*, 154104.

(3) Perdew, J. P.; Burke, K.; Ernzerhof, M. Generalized Gradient Approximation Made Simple. *Phys. Rev. Lett.* **1996**, *77*, 3865–3868.

(4) Perdew, J. P.; Burke, K.; Ernzerhof, M. Generalized Gradient Approximation Made Simple [Phys. Rev. Lett. 77, 3865 (1996)]. *Phys. Rev. Lett.* **1997**, *78*, 1396–1396.

(5) Grimme, S.; Ehrlich, S.; Goerigk, L. Effect of the Damping Function in Dispersion Corrected Density Functional Theory. *J. Comput. Chem.* **2011**, *32*, 1456–1465.

(6) van Lenthe, E.; Baerends, E. J. Optimized Slater-Type Basis Sets for the Elements 1–118. *J. Comput. Chem.* **2003**, *24*, 1142–1156.

(7) Ziegler, T.; Rauk, A. A Theoretical Study of the Ethylene-Metal Bond in Complexes Between Cu^+, Ag^+, Au^+, Pt^0, or Pt^{2+} and Ethylene, Based on the Hartree-Fock-Slater Transition-State Method. *Inorg. Chem.* **1979**, *18*, 1558–1565.

(8) Ziegler, T.; Rauk, A. CO, CS, N_2, PF_3, And $CNCH_3$ as σ Donors and π Acceptors. a Theoretical Study by the Hartree-Fock-Slater Transition-State Method. *Inorg. Chem.* **1979**, *18*, 1755–1759.

(9) Mitoraj, M. P.; Michalak, A.; Ziegler, T. A Combined Charge and Energy Decomposition Scheme for Bond Analysis. *J. Chem. Theory Comput.* **2009**, *5*, 962–975.

(10) van Lenthe, E.; Baerends, E. J.; Snijders, J. G. Relativistic Total Energy Using Regular Approximations. *J. Chem. Phys.* **1994**, *101*, 9783–9792.

(11) *Gaussian 16. A. 01.* Frisch, M. J.; Trucks, G. W.; Schlegel, H. B.; Scuseria, G. E.; Robb, M. A.; Cheeseman, J. R.; Scalmani, G.; Barone, V.; Petersson, G. A.; Nakatsuji, H.; Li, X.; Caricato, M.; Marenich, A. V.; Bloino, J.; Janesko, B. G.; Gomperts, R.; Mennucci, B.; Hratchian, H. P.; Ortiz, J. V.; Izmaylov, A. F.; Sonnenberg, J. L.; Williams-Young, D.; Ding, F.; Lipparini, F.; Egidi, F.; Goings, J.; Peng, B.; Petrone, A.; Henderson, T.; Ranasinghe, D.; Zakrzewski, V. G.; Gao, J.; Rega, N.; Zheng, G.; Liang, W.; Hada, M.; Ehara, M.; Toyato, K.; Fukuda, R.; Hasegawa, J.; Ishida, M.; Nakajima, T.; Honda, Y.; Kitao, O.; Nakai, H.; Vreven, T.; Throssell, K.; Montgomery, J. A. ,. J.; Peralta, J. E.; Ogliaro, F.; Bearpark, M. J.; Heyd, J. J.; Brothers, E. N.; Kudin, K. N.; Staroverov, V. N.; Keith, T. A.; Kobayashi, R.; Normand, J.; Raghavachari, K.; Rendell, A. P.; Burant, J. C.; Iyengar, S. S.; Tomasi, J.; Cossi, M.; Millam, J. M.; Klene, M.; Adamo, C.; Cammi, R.; Ochterski, J. W.; Martin, R. L.; Morokuma, K.; Farkas, O.; Foresman, J. B.; Fox, D. J. Gaussian, Inc.: Wallingford CT **2016**.

(12) Ernzerhof, M.; Scuseria, G. E. Assessment of the Perdew–Burke–Ernzerhof Exchange-Correlation Functional. *J. Chem. Phys.* **1999**, *110*, 5029–5036.

(13) Adamo, C.; Barone, V. Toward Reliable Density Functional Methods Without Adjustable Parameters: the PBE0 Model. *J. Chem. Phys.* **1999**, *110*, 6158–6170.

(14) Hay, P. J.; Wadt, W. R. Ab Initio Effective Core Potentials for Molecular Calculations. Potentials for the Transition Metal Atoms Sc to Hg. *J. Chem. Phys.* **1985**, *82*, 270–283.

(15) Hay, P. J.; Wadt, W. R. Ab Initio Effective Core Potentials for Molecular Calculations. Potentials for K to Au Including the Outermost Core Orbitals. *J. Chem. Phys.* **1985**, *82*, 299–310.

(16) Petersson, G. A.; Bennett, A.; Tensfeldt, T. G.; Laham, Al, M. A.; Shirley, W. A.; Mantzaris, J. A Complete Basis Set Model Chemistry. I. the Total Energies of Closed-Shell Atoms and Hydrides of the First-Row Elements. *J. Chem. Phys.* **1988**, *89*, 2193–2218.

(17) Lu, T.; Chen, F. Multiwfn: a Multifunctional Wavefunction Analyzer. *J. Comput. Chem.* **2011**, *33*, 580–592.
